# Facilitators and Barriers to Implementing AI in Routine Medical Imaging: Systematic Review and Qualitative Analysis

**DOI:** 10.2196/63649

**Published:** 2025-07-21

**Authors:** Katharina Wenderott, Jim Krups, Matthias Weigl, Abigail R Wooldridge

**Affiliations:** 1 Institute for Patient Safety University Hospital Bonn Bonn Germany; 2 Department of Industrial and Enterprise Systems Engineering University of Illinois Urbana-Champaign Urbana, IL United States

**Keywords:** artificial intelligence, medical imaging, work system barriers and facilitators, implementation science, sociotechnical system, systems analysis, ergonomics, workflow, Systems Engineering Initiative for Patient Safety, SEIPS

## Abstract

**Background:**

Artificial intelligence (AI) is rapidly advancing in health care, particularly in medical imaging, offering potential for improved efficiency and reduced workload. However, there is little systematic evidence on process factors for successful AI technology implementation into clinical workflows.

**Objective:**

This study aimed to systematically assess and synthesize the facilitators and barriers to AI implementation reported in studies evaluating AI solutions in routine medical imaging.

**Methods:**

We conducted a systematic review of 6 medical databases. Using a qualitative content analysis, we extracted the reported facilitators and barriers, outcomes, and moderators in the implementation process of AI. Two reviewers analyzed and categorized the data separately. We then used epistemic network analysis to explore their relationships across different stages of AI implementation.

**Results:**

Our search yielded 13,756 records. After screening, we included 38 original studies in our final review. We identified 12 key dimensions and 37 subthemes that influence the implementation of AI in health care workflows. Key dimensions included evaluation of AI use and fit into workflow, with frequency depending considerably on the stage of the implementation process. In total, 20 themes were mentioned as both facilitators and barriers to AI implementation. Studies often focused predominantly on performance metrics over the experiences or outcomes of clinicians.

**Conclusions:**

This systematic review provides a thorough synthesis of facilitators and barriers to successful AI implementation in medical imaging. Our study highlights the usefulness of AI technologies in clinical care and the fit of their integration into routine clinical workflows. Most studies did not directly report facilitators and barriers to AI implementation, underscoring the importance of comprehensive reporting to foster knowledge sharing. Our findings reveal a predominant focus on technological aspects of AI adoption in clinical work, highlighting the need for holistic, human-centric consideration to fully leverage the potential of AI in health care.

**Trial Registration:**

PROSPERO CRD42022303439; https://www.crd.york.ac.uk/PROSPERO/view/CRD42022303439

**International Registered Report Identifier (IRRID):**

RR2-10.2196/40485

## Introduction

### Background

Advancements in the development of artificial intelligence (AI) have increased the accessibility and awareness of AI solutions in health care [1,2]. AI in health care has numerous potential applications, which can be categorized into 4 areas of application: diagnostics, therapeutics, administration and regulation, and population health management [3]. AI is mostly applied to data-driven tasks due to its ability to adapt to input data. It can process and analyze large volumes of health care data more quickly [4,5].

In the United States and Europe, AI technologies in health care can be categorized as software as a medical device, referring to software designed for medical purposes without requiring hardware integration [6]. These purposes, as defined by the Food and Drug Administration, encompass treating, diagnosing, curing, mitigating, or preventing diseases or conditions [7]. The growing recognition of the potential of AI algorithms in health care is supported by the surge of Food and Drug Administration approvals since 2016 for AI-enabled devices [8]. Notably, >75% of approvals are related to radiology [8]. These numbers are consistent with reports that highlight image-based disciplines at the forefront of AI integration in clinical practice due to their data-driven nature and continuously increasing workload demands [3,5,9].

Despite the increasing availability of AI algorithms, there remains a limited understanding of their integration into clinical practice. A critical gap persists between broad research on algorithm development and limited evaluation of their actual use in clinical practice [10,11]. Most AI solutions are tested under controlled experimental conditions, which may underestimate the real-world impact of contextual factors on their utility and are therefore not necessarily transferable to clinical applications [12]. Depending on the users, the implementation process, and the clinical setting, the usefulness of AI solutions can significantly differ from previous evaluations or applications [13,14].

Complex sociotechnical systems, such as health care, “can be characterised by high uncertainty, multiple interacting elements and dynamic change” [15]. According to the sociotechnical systems theory, a sociotechnical system refers to the integration of humans, machines, environments, and organizational processes working together toward a shared objective. It consists of 2 interconnected subsystems: the technology subsystem, which encompasses tools and work organization, and the social subsystem, which involves individuals, teams, and coordination needs [15,16]. Sociotechnical frameworks of real-world clinical care offer a valuable approach to scrutinizing implementation complexities as well as the multiple intricacies of technology adoption [17,18].

A framework based on the sociotechnical systems theory that captures these complex demands and relations in health care settings is the Systems Engineering Initiative for Patient Safety (SEIPS) model [17]. The SEIPS model—most recently refined as SEIPS 3.0 [19]—proposes that sociotechnical systems consist of 5 interacting components: people, tasks, tools and technologies, organization, and environment. When one of the components changes, it affects the other components of the work system and subsequently the outcomes, that is, for patients, health care professionals, or organizations [17]. The model emphasizes the human as the center of the work system, which should be designed to support human performance and minimize negative impacts resulting from the work setting [17,19]. The SEIPS model can be applied to identify barriers and facilitators, which result from 1 element or the interaction between elements [20]. Hoonakker et al [21] introduced the concept of dimensions, which can function as either facilitators or barriers.

While the SEIPS model is useful for understanding work system dynamics, other frameworks also help analyze health care technology implementation. The Consolidated Framework for Implementation Research (CFIR) evaluates implementation processes in health services through 5 domains: intervention characteristics, outer setting, inner setting, individual characteristics, and the implementation process, overlapping with SEIPS in addressing the involved people and their environment [22,23]. The nonadoption, abandonment, scale-up, spread, and sustainability (NASSS) framework examines factors influencing each of these outcomes and is specifically designed for technology implementation, while SEIPS covers broader work system design [24,25]. The integrate, design, assess, and share (IDEAS) framework, focusing on the full development cycle, is more suited for creating health technology solutions but less relevant to our study, which focuses on evaluating already implemented AI solutions [26]. The key distinction of SEIPS 3.0 is its human-centered approach, placing patients, clinicians, and caregivers at the core of the work system and emphasizing human-technology interaction and alignment in real-world clinical environments [19].

A thorough understanding of how professionals in real-world clinical settings use AI technologies and how these tools can support their performance seems imperative, given the increasing availability of AI in health care [27]. While current literature extensively addresses the potential of AI in overviews and opinion articles, limited empirical evidence stems from actual clinical care [11,28-30]. This leads to a critical lack of comprehensive understanding of AI implementation challenges and processes, potentially limiting the future development of evidence-based recommendations for successful AI technology implementation in clinical practice.

### Objectives

Given the growing number of AI solutions in imaging-based disciplines, we aimed to explore and synthesize the existing literature on facilitators and barriers to AI implementation in routine medical imaging. We explored the relationships among AI implementation factors by drawing upon the SEIPS model. This approach allows for a concept-based and comprehensive synthesis of the available literature, generating a nuanced understanding of key process facilitators and barriers and their interactions in the implementation of AI technology into sociotechnical work systems in health care. Moreover, it contributes to a holistic picture of AI implementation in clinical work with consideration of important outcomes and moderating factors.

## Methods

### Registration and Protocol

Before starting, we registered our systematic literature review, which included qualitative analysis and synthesis, in the PROSPERO database (CRD42022303439) and published the review protocol (RR2-10.2196/40485) [28].

The primary aim of this study was to assess and synthesize facilitators and barriers to AI workflow integration in medical imaging. This study was part of a larger review project on the impact of AI solutions on workflow efficiency in medical imaging, with a separate publication on the effect of AI on efficiency outcomes [31]. Our report follows the PRISMA (Preferred Reporting Items for Systematic Reviews and Meta-Analyses) reporting guidelines (Multimedia Appendix 1).

### Eligibility Criteria

We analyzed original clinical imaging studies in German or English published in peer-reviewed journals from January 2000 onward. Eligible studies implemented AI into real-world clinical workflows; therefore, we included observational and interventional studies (eg, randomized controlled trials) conducted in health care facilities using medical imaging. We focused on AI tools interpreting image data for disease diagnosis and screening.

We excluded dissertations, conference proceedings, and gray literature. In addition, due to our focus on real-world implementation of AI, we excluded studies conducted in experimental or laboratory settings.

### Search Strategy

We searched the following electronic databases: MEDLINE (PubMed), Embase, PsycINFO, Web of Science, IEEE Xplore, and Cochrane CENTRAL. The databases were selected to reflect the interdisciplinary research on AI implementation in health care by including sources from medicine, psychology, and IT. Databases such as Cochrane, which only list systematic reviews or meta-analyses, were excluded in accordance with our eligibility criteria.

The detailed search strategy followed the PICO (population, intervention, comparison, and outcome) framework and can be found in the study by Wenderott et al [31]. The searches were performed on July 21, 2022, and on May 19, 2023. In a backward search, we identified additional relevant studies through screening the references of the included studies from the database search. Due to the time-consuming process of a systematic review with the in-depth qualitative analysis of the included studies, we performed an additional search on November 28, 2024, to identify relevant, recently published studies on facilitators and barriers to AI implementation in medical imaging [32]. This additional step ensured an update as well as the incorporation of interim published evidence on the topic. Further details are provided in Multimedia Appendix 2 [29,33-40].

### Screening and Selection Procedure

All gathered articles were imported into the Rayyan tool (Rayyan) [41] for initial title and abstract screening. Two study team members (KW plus JK, MW, or Nikoloz Gambashidze), trained beforehand, individually assessed the titles and abstracts and reviewed their decisions in a consensus-oriented discussion. Subsequently, KW and JK screened the full texts of all eligible publications. Any disagreements regarding article inclusion were resolved through discussions with a third team member (MW). Exclusion reasons were documented and presented a flow diagram [42].

### Data Extraction

For qualitative data extraction, full texts of all eligible articles were imported into MAXQDA 22 (VERBI Software GmbH) [43]. This program allows users to mark text segments with different semantic codes, in this case the key characteristics, and automatically creates Excel (Microsoft Corporation) files of all the marked segments. Two researchers (JK and Fiona Zaruchas) extracted key study characteristics, including country, sample size, and any reported conflicts of interest (for more details, refer to the study protocol [28]). Countries and authors were imported into RStudio (2025.05.1+513; Posit PBC) to create a map of the geographical distribution [44].

Regarding the reported stage and status of AI tool implementation in clinical practice, we used the studies by Bertram et al [45] and Pane and Sarno [46] to develop our classification of “level of implementation.” We defined 3 distinct levels: external validation, initial implementation, and full implementation (Textbox 1). We categorized all the included studies accordingly.

**Levels of artificial intelligence (AI) implementation in clinical practice.**

**External validation**

- Evaluation of the AI solution using real-world data
- Participants (ie, clinicians) recruited for the study
- Participants potentially blinded to other patient data
- Approximate simulation of the routine workflow

**Initial implementation**

- Partial implementation into the usual workflow
- Participants recruited in their usual work
- Different study groups possible

**Full implementation**

- Used for all eligible patients
- Implemented into the routine workflow of clinicians

### Data Analysis

We applied a multistep procedure for data analysis. We first used a structured qualitative content analysis in a stepwise process [47]. In the initial phase, JK and KW independently classified the following key content categories of AI technology process factors in all the retrieved study texts:

- Facilitators, defined as “any factor that promotes or expands the integration or use of the AI system in the workflow” [48].
- Barriers, defined as “any factor that limits or restricts the integration or use of the AI system” [48].
- Outcomes of AI use, defined as the impact the AI use has on clinicians, patients, organizations, or the workflow.
- Moderators, defined as external factors, independent of the AI tool, that influence its use, for example, the setting or user [33].

Subsequently, JK and KW engaged in a consensus-oriented discussion to reconcile all coded text segments [47,49]. In the following step, we defined subcategories following an inductive process. We noted a thematic overlap between topics being reported as a facilitator or barrier, depending on the study. Therefore, we decided to code categories that encompass facilitators as well as barriers, noting their valence (ie, positive or negative) separately. We organized the categories in a comprehensive codebook with corresponding definitions [47]. To establish consistency between raters throughout the coding process, the codebook underwent testing across 5 publications, where we discussed any coding issues and adjusted definitions as needed. Moving forward, both researchers (KW and JK) independently coded segments and subsequently discussed their codes to establish a consensus. Two researchers (KW and ARW) independently identified the proximally involved work system elements of the dimensions and then met to discuss their categorization and reached a consensus [20,50]. Using an inductive methodology, individual statements per dimension were clustered into themes that were mentioned frequently.

### Epistemic Network Analysis

Epistemic network analysis (ENA) examines relationships between codes by modeling how frequently they co-occur in datasets. ENA was developed, validated, and widely applied in engineering education studies and has subsequently been used in research focused on human factors in health care [51-56]. ENA quantifies qualitative data by applying mathematics similar to social network analysis and principal component analysis to generate a weighted network of co-occurrences of codes. The matrix is then depicted graphically for each unit within the dataset. In each graph, the node size represents how frequently a code occurred in that unit; the thickness of the edges between the nodes corresponds to the weight, or frequency, at which a pair of codes co-occurred. The placement of each node is based on plotting vectors from the weighted co-occurrence matrix in a high-dimensional space, normalizing the vectors, reducing the dimensions using singular value decomposition (similar to principal component analysis), and then performing a rigid body rotation to preserve meaning. The x-axis is the dimension that accounts for the highest variation in the dataset, and the y-axis is a dimension orthogonal to the first that explains the next highest percentage of variance. Due to the preservation of meaning, these dimensions can be interpreted conceptually based on the qualitative data analysis. The fit of the resulting model can be evaluated both with Spearman and Pearson correlation coefficients. Importantly, ENA evaluates all networks concurrently, yielding a collection of networks that can be compared both visually and statistically. For more details on the method, including the mathematics and validation, please refer to the studies by Andrist et al [57], Bowman et al [58], Shaffer [59], Shaffer et al [56], and Shaffer and Ruis [60].

ENA serves as a valuable method to analyze and visualize the findings of our qualitative content analysis, that is, the co-occurrence of the dimensions of facilitators or barriers in the included studies [56,58-60]. In this study, we used the ENA web tool (version 1.7.0) [61]. The data were uploaded to the ENA web tool in a .csv file, with each row representing a barrier or facilitator identified through qualitative analysis; the columns included metadata such as the study, type of implementation, if that row contained a barrier or a facilitator, the dimension that specific barrier or facilitator was categorized as, and the coded excerpt from the study. ENA was used to generate 6 network graphs that depict the relationships between barriers or facilitators reported in each study, separated by the level of implementation. Thus, in each graph, the node size corresponds to the frequency that a barrier or facilitator occurred across all studies in that type of implementation; the thickness of the edges between nodes indicates how often a pair of barriers or facilitators co-occurred within the same study.

## Results

### Study Selection

**Figure 1:**
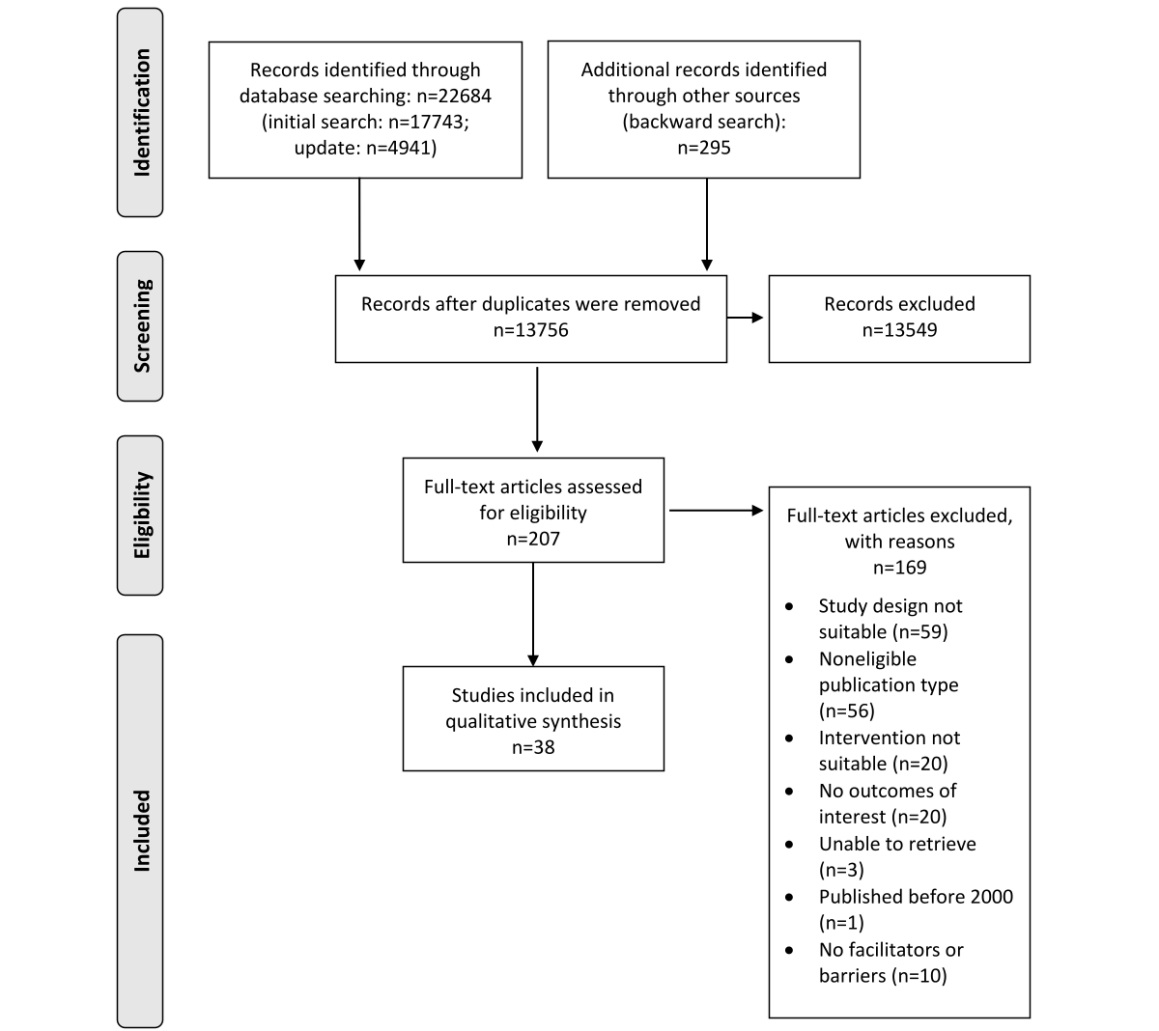
PRISMA (Preferred Reporting Items for Systematic Reviews and Meta-Analyses) flowchart.

We identified 22,684 records in the databases and an additional 295 articles through a backward search. After the removal of duplicates, 13,756 remaining records were included in the title and abstract screening. Afterward, 207 full texts were screened, of which 169 were excluded primarily because they did not meet the inclusion criteria, that is, experimental studies or studies not focusing on AI tools for interpreting imaging data (for more details, refer to the study by Wenderott et al [28]). A total of 10 studies were excluded because they did not describe any facilitator or barrier in the course of clinical implementation. Finally, 38 studies were included in the review and data extraction. A PRISMA flowchart is presented in Figure 1.

### Study Characteristics

**Figure 2:**
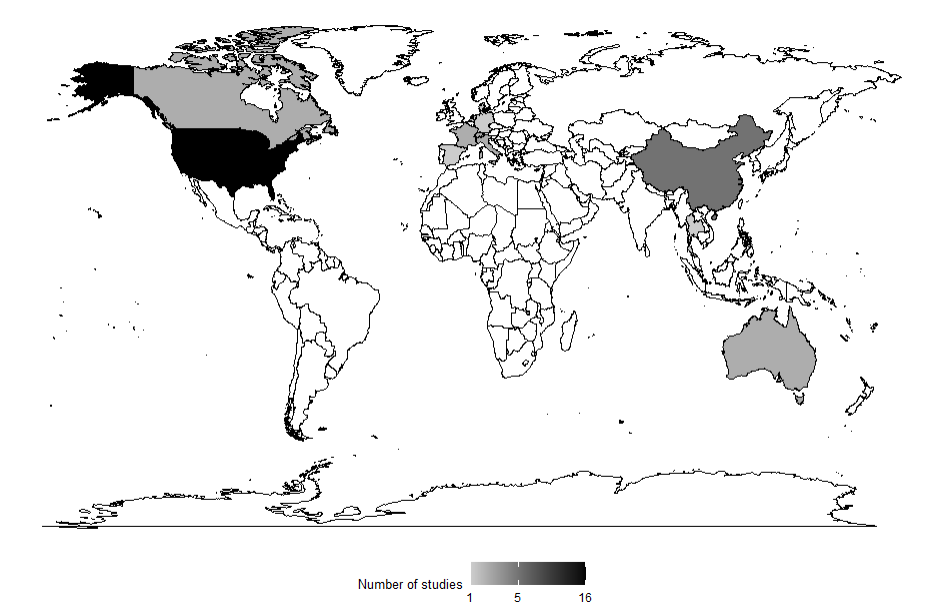
Geographical distribution of the included studies (created with RStudio).

Of the 38 included studies, 24 (63%) were performed in a single institution and 14 (37%) were multicenter studies. Only 5% (2/38) of the studies were published before 2012, whereas all others (36/38, 95%) were published from 2018 onward. The geographical distribution of the studies is depicted in Figure 2. On the basis of the heterogeneity in the regulatory frameworks of AI in health care, we included a comparison across dimensions between the 2 main geographical clusters, the European Union and the United States (Multimedia Appendix 3 [62-64]). Most studies (25/38, 66%) were conducted in radiology, followed by gastroenterology (5/38, 13%; Table 1). A total of 47% (18/38) of the studies reported a potentially relevant conflict of interest. For the risk of bias assessment, we used the Risk of Bias in Nonrandomized Studies of Interventions tool and the Cochrane Risk of Bias version 2 tool for the 1 included randomized study [65,66]. From the included 37 nonrandomized studies, only 1 (3%) study was classified as having a low risk of bias. In total, 11% (4/37) of the studies were rated as having a moderate risk, 65% (24/37) of the studies had a serious risk, and 22% (8/37) of the studies were assessed as having a critical risk of bias. The included randomized study was determined to have a high overall risk of bias. For a detailed risk of bias and quality of reporting assessment, refer to the supplementary material of the study by Wenderott et al [31].

**Table 1:** Reported key characteristics of the included studies.

Study	Data collection	Source of data	Professionals, n	Cases, patients, or scans, n	Level of implementation
Arbabshirani et al [67]	Prospective	No information	Radiologists (not specified)	347 patients	Full
Batra et al [68]	Retrospective	Time stamps	32 radiologists	2501 examinations of 2197 patients	Full
Carlile et al [69]	Prospective	Survey	112 ED^a^ physicians	1855 scans and a survey on 202 scans	Initial
Cha et al [70]	Prospective	Survey	18 physicians	173 patients	Full
Cheikh et al [71]	Retrospective	Performance metrics and survey	79 radiologists	7323 examinations	Initial
Chen et al [72]	Retrospective	Performance metrics and time measurement	4 radiologists	85 patients	External
Conant et al [73]	Retrospective	Performance metrics and time measurement	24 radiologists (including 13 breast subspecialists)	260 cases	External
Davis et al [74]	Prospective	Time stamps	Radiologists (not specified)	50,654 cases	Full
Diao et al [75]	Prospective	Time stamps and survey	7 radiologists	251 patients	Initial
Duron et al [76]	Retrospective	Performance metrics and time stamps	6 radiologists and 6 ED physicians	600 cases	External
Elijovich et al [77]	Retrospective	Chart review	Neurologists and neurointerventionalists (not specified)	680 patients	Full
Ginat [78]	Retrospective	Time stamps	5 radiologists	8723 scans	Initial
Hassan et al [79]	Retrospective	Chart review	Technologists, radiologists, ED physicians, neurologists, and interventionalists (not specified)	63 patients	Full
Jones et al [80]	Prospective	Survey	11 radiologists	2972 scans of 2665 patients	Initial
Ladabaum et al [81]	Retrospective	Chart review	52 endoscopists	2329 patients	Initial
Levy et al [82]	Retrospective	Performance metrics and time stamps	30 gastroenterologists	4414 patients	Full
Marwaha et al [83]	Retrospective	Survey	Genetic counselors and trainees (15 in total)	72 patients	Initial
Mueller et al [84]	Prospective	Observation, interview, and survey	2 radiologists	90 scans	Full
Nehme et al [85]	Prospective	Performance metrics, time stamps, and surveys	Endoscopists and staff members (45 in total)	1041 patients	Initial
Oppenheimer et al [86]	Prospective	Performance metrics	2 radiologists	1163 examinations of 735 patients	Full
Pierce et al [87]	Retrospective	Case review	Radiologists (not specified)	30,847 examinations	Full
Potrezke et al [88]	Prospective	Performance metrics	49 radiologists and 12 medical image analysts	170 cases of 161 patients	Initial
Quan et al [89]	Prospective	Performance metrics and time measurement	6 endoscopists	600 patients	Full
Raya-Povedano et al [90]	Retrospective	Performance metrics and workload	5 breast radiologists	15,986 patients	External
Ruamviboonsuk et al [91]	Prospective	Performance metrics and surveys	Staff members and nurses (12 in total)	7651 patients	Full
Sandbank et al [92]	Prospective	Performance metrics	Pathologists (not specified)	5954 cases	Full
Schmuelling et al [93]	Retrospective	Performance metrics and time stamps	Radiologists (not specified)	1808 scans of 1770 patients	Full
Seyam et al [94]	Retrospective	Performance metrics and time stamps	Radiologists (not specified)	4450 patients	Full
Tchou et al [95]	Prospective	Observation	5 radiologists	267 cases	External
Tricarico et al [96]	Prospective	Performance metrics	Radiologists (not specified)	2942 scans	Initial
Vassallo et al [97]	Retrospective	Observation and performance metrics	3 radiologists	225 patients	External
Wang et al [98]	Prospective	Performance metrics and time measurement	8 endoscopists	1058 patients	External
Wang et al [99]	Retrospective	Chart review	2 radiologists	2120 patients	External
Wittenberg et al [100]	Retrospective	Performance metrics and time measurement	6 radiologists	209 patients	External
Wong et al [101]	Prospective	Survey	Radiation therapists and oncologists (39 in total)	174 cases	Full
Wong et al [102]	Prospective	Performance metrics and survey	Radiologists and internists (17 in total)	214 scans	Initial
Yang et al [103]	Prospective	Performance metrics and time measurement	Ophthalmologists	1001 patients	Initial
Zia et al [104]	Prospective	Performance metrics, time stamps, and survey	49 radiologists	1446 scans	Initial

^a^ED: emergency department.

Regarding the level of AI implementation, we identified 24% (9/38) of the studies evaluating external validation, 34% (13/38) of the studies focusing on initial implementation, and 42% (16/38) of the studies focusing on an AI tool being fully integrated in the clinic. Table 1 presents the key characteristics of all the included studies. There was a substantial variety of AI technologies, with 42% (16/38) of the studies using commercial AI solutions and 55% (21/38) of the studies evaluating self-developed tools (1 study did not specify the source of the AI solution [87]). More details about the AI tools are provided in Multimedia Appendix 4 [67-104]. The methods that were most frequently used were the analysis of performance metrics (21/38, 55%) or time stamps (10/38, 26%). In total, 29% (11/38) of the studies used some form of survey or questionnaire to gather the opinions and experiences of clinicians. Most commonly, they used self-reports on the impact of AI use on the diagnosis and efficiency, followed by their attitude toward AI, their satisfaction or usefulness, as well as the usability of the AI tool. Notably, only the study by Jones et al [80] used an established tool, that is, the Systems Usability Scale. Further details on the surveys described in the studies are provided in Multimedia Appendix 5 [69,71,75,80,83-85,91, 101,102,104].

### Facilitators and Barriers to AI Implementation

#### Identification and Classification of Process Factors (Qualitative Content Analysis Results)

##### Overview

**Figure 3:**
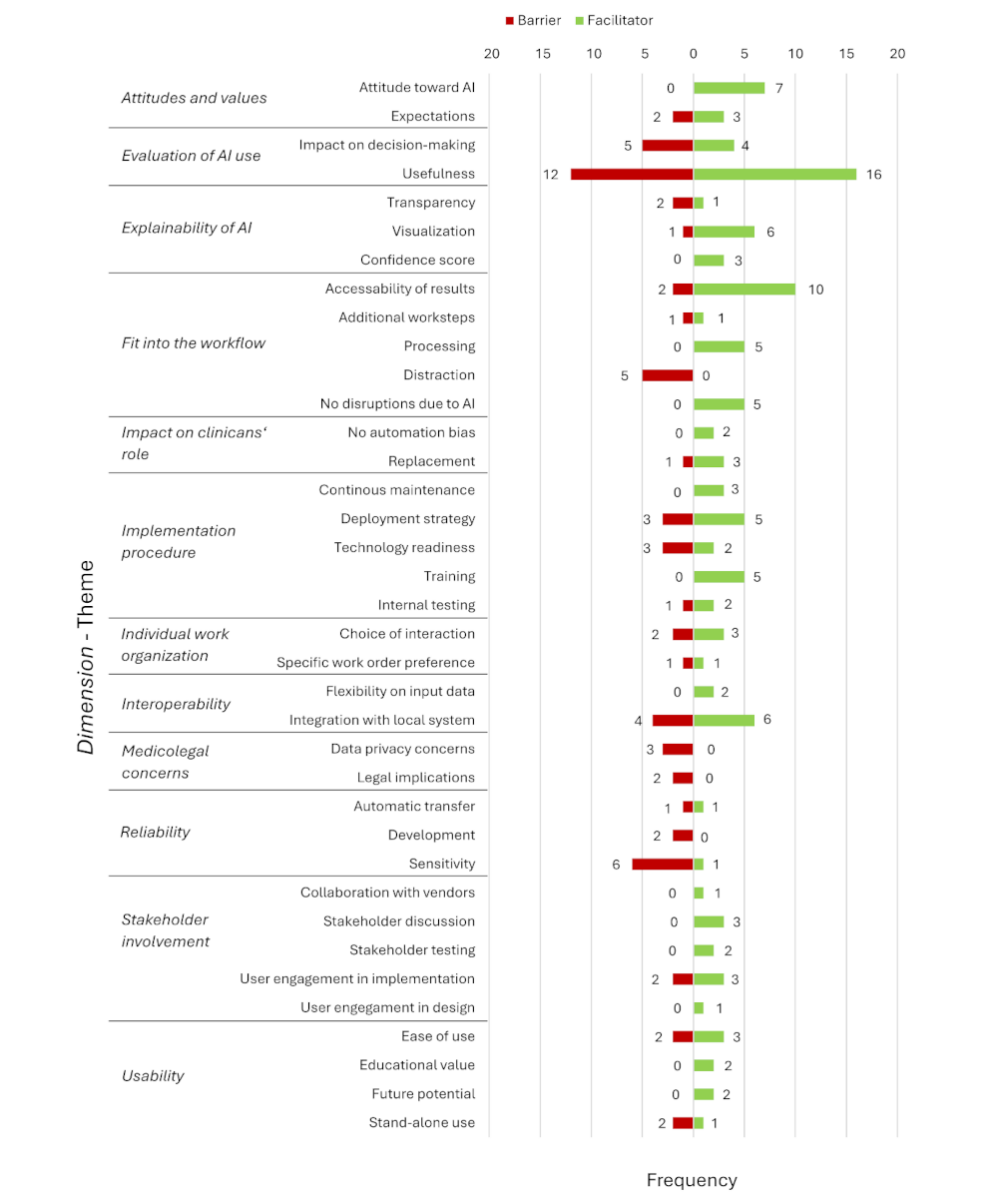
Themes of reported facilitators and barriers to the implementation of artificial intelligence (AI) in medical imaging.

Drawing upon the qualitative analyses of the included studies, we identified 180 statements from the included publications that described the factors influencing AI implementation in clinical practice. These statements were systematically categorized into 12 overarching dimensions, as described in detail in Table 2. Within each dimension, we clustered recurring themes. This resulted in a total of 37 themes; the details and example quotations from the studies are listed in Multimedia Appendix 6 [67-104]. Many themes were stated simultaneously as facilitators and barriers, mostly depending on the presence or absence of the mentioned theme in the study (Figure 3). For example, the theme *impact on decision-making* was referenced positively in the study by Cheikh et al [71]:

Radiologists stressed the importance of AI to strengthen their conclusions, especially to confirm negative findings, or to ensure the absence of distal PE [pulmonary embolism] in poor-quality examinations.

In contrast, Oppenheimer et al [86] stated the following:

In some edge cases, both residents reported feeling somewhat unsure of their diagnosis, in particular if they decided on a fracture and the AI result was negative.

With 64% (115/180) of the segments, we identified more facilitators in general than barriers (65/180, 36% segments). The dimensions *attitudes and values* and *stakeholder engagement* were mostly stated as facilitators, highlighting their positive impact on AI implementation. *Medicolegal concerns* was the only dimension that was exclusively mentioned as a barrier. In the subsequent sections, we describe the 3 dimensions with the most frequently coded segments in more detail.

**Table 2:** Dimensions of facilitators and barriers to artificial intelligence (AI) implementation, including definitions and examples.

Dimensions	Definition	Codes, n	Work system elements					
			People	Tasks	TT^a^	Organization	PE^b^	EE^c^
Evaluation of AI use	Clinicians’ or patients’ evaluation of the usefulness of the AI tool impacting its integration.	37	✓	✓	✓			
Fit into the workflow	The AI is embedded into the workflow or processes of the local health care facility, including both clinical workflows and technical aspects such as data processing.	29		✓	✓	✓		
Implementation procedure	The AI implementation follows an implementation protocol or a prespecified plan, including users receiving training on the AI tool.	24	✓		✓	✓		
Explainability of AI	The capability of understanding and justifying the decisions made by the AI tool.	13			✓			
Attitudes and values	The beliefs, ethical principles, judgments, or priorities that might have been present before using AI influence clinicians’ acceptance, adoption, and use of AI.	12	✓		✓			
Interoperability	Ensures that AI can seamlessly communicate and share data with other technologies used.	12			✓	✓		
Stakeholder involvement	In the course of implementing or using AI, important stakeholders are included in the process.	12	✓	✓	✓	✓		
Usability	Users can interact effectively and intuitively with the AI tool to accomplish their goals.	12		✓	✓			
Reliability	The reliability of the AI tool that impacts its use in the workflow.	11			✓			
Individual work organization	Fit of the AI tool with the individual preferences of the users’ work organization.	7	✓	✓	✓			
Impact on the role of clinicians	AI use alters the role of clinicians, how they perceive autonomy, and whether they feel responsible for their diagnosis.	6	✓	✓	✓			
Medicolegal concerns	Intersection of medical practice and legal regulations, mitigation of legal risks, and safeguarding of patients and their rights when using the AI tool.	5			✓	✓		✓

^a^TT: tools and technologies.

^b^PE: physical environment.

^c^EE: external environment.

##### Evaluation of AI Use

The dimension *evaluation of AI use* reflected whether a positive or negative evaluation of the use of the AI solution aided the AI integration. This dimension was most frequently mentioned, reflecting the focus of the included studies on AI evaluation in clinical practice. We identified *people*, *tasks*, and *tools and technologies* as proximally involved work system elements. Two themes emerged in this dimension. Overall, the *usefulness* was the most frequently mentioned theme. This is supported by evidence that perceived usefulness or performance expectancy are strong determinants of the actual use of technologies [105,106], focusing on the behavior of users. The *impact on decision-making* emerged as a second theme in this dimension. Positively, clinicians valued the support provided by the AI tool, as AI use can increase the confidence of clinicians [107]. Negatively, the studies mentioned risks, such as alert fatigue [104], over trust [81,82], or insecurities due to diverging diagnostic decisions [86].

##### Fit Into the Workflow

The dimension *fit into the workflow* focused on how well AI technology fits into the workflow, which is an important factor to consider during the implementation of a novel technology [108,109]. The proximally involved work system elements were *tasks*, *tools and technologies*, and *organization*. In this dimension, 5 themes were identified. The most frequently and favorably mentioned theme was the *accessibility of results*, for example, by results being forwarded automatically to the clinicians [77] or providing a notification platform [78]. This also applied to the theme of *data processing,* where automatic and fast processing was a facilitating factor [67,68,77,78,97]. Regarding the themes distractions or disruptions due to AI, the facilitating factors were characterized by the absence of these, whereas the barriers reflected the negative influence of the AI tool on the workflow of the users, for example, through alarms that potentially distracted the clinicians. The theme *additional work steps* was only mentioned in the study by Batra et al [68].

##### Implementation Procedure

The dimension *implementation procedure* focused on the descriptions of the implementation process to install the AI system in the clinical workflow. The related work system elements were *people*, *tools and technologies*, and *organization*. In this dimension, the themes *internal testing* of the AI tool; *continuous maintenance*, that is, the ongoing monitoring of the AI tool with adaptations if necessary; and the *training of users* were exclusively mentioned as facilitators. Of the 38 studies, only 3 (8%) described a *deployment strategy* [81,87,88], with Ladabaum et al [81] describing that their minimalist approach was not sufficient to successfully implement the AI tool. In total, 13% (5/38) of the studies discussed the strategies or preconditions to the *technology readiness* of the organization, which can be defined as the willingness to “embrace and use new technologies to accomplish goals.... It is a combination of positive and negative technology-related beliefs” [110]. In the study by Ruamviboonsuk et al [91], the authors encountered the challenge that the hospital was still working with paper-based records, and the internet connectivity was slow, highlighting the role of the pre-existing digital infrastructure.

#### Comparison of Facilitators and Barriers Across the Levels of Implementation (Results of ENA)

**Figure 4:**
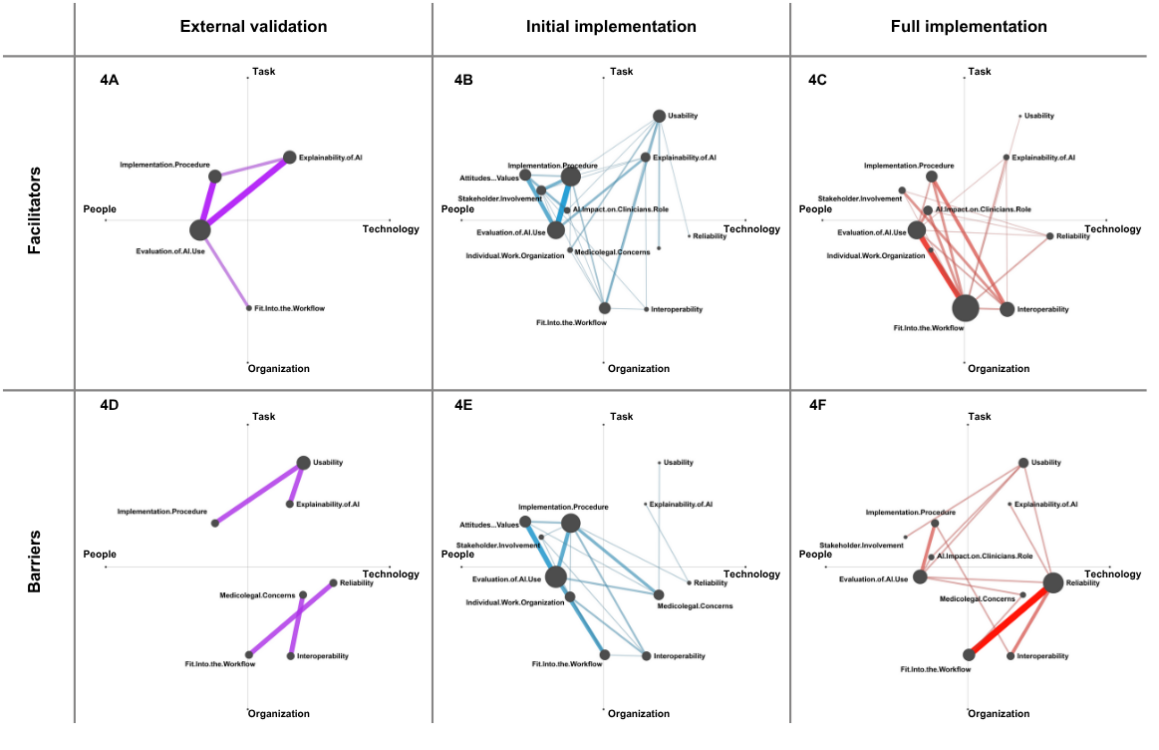
Facilitators and barriers to artificial technology (AI) technology implementation in medical imaging: network diagrams resulting from an epistemic network analyses separated by the level of implementation.

We used ENA to model the differences in facilitators and barriers across the level of implementation, resulting in 6 distinct network graphs (Figure 4). The axes identified in our ENA can be associated with work system elements of the SEIPS model [17]. The x-axis represents the work system element *people* in the negative direction, as indicated by the dimensions *attitudes and values* and *stakeholder involvement* being the farthest in this direction, and the work system element *technology* in the positive direction, which we concluded from the dimensions *reliability*, *interoperability,* and *usability* presented in this direction. For the x-axis and the y-axis, the coregistration correlations were 1 (both Pearson and Spearman), showing a strong goodness of fit [111]. The x-axis accounted for 37.2% of the variance. The y-axis accounted for 21% of the variance. The positive direction of the y-axis can be associated with the work system element *tasks*, with the ENA showing the dimension *usability* as the farthest node in this direction. In contrast, the negative side of the y-axis represents the work system element *organization*, which we inferred from the dimensions *fit into the workflow* and *interoperability* being the most distant nodes in this direction.

For the studies describing external validations of AI solutions, a total of 19 coded segments (segments per study: mean 2.11, SD 1.27; median 2, IQR 1-2) were included in the ENA. The resulting networks showed a small number of involved dimensions and connections, highlighting the dimensions *evaluation of AI use* and *explainability of AI* as facilitators and the dimension *usability* as a barrier (Figures 4A and 4D).

For the initial implementation studies, we analyzed 85 coded segments (segments per study: mean 6.54, SD 4.74; median 5, IQR 3-9). The facilitators showed an accumulation in the quadrant of the work system elements *tasks* and *people*, with the dimensions *implementation procedure* and *evaluation of AI use* being the largest nodes. The strongest connection for the facilitators was between the dimensions *evaluation of AI use* and *implementation procedure*, whereas the strongest connection for the barriers was between the dimensions *evaluation of AI use* and *attitudes and values*, with the dimension *implementation procedure* being also mentioned frequently (Figures 4B and 4E).

Regarding the publications reporting the full implementation of AI solutions, the network graphs were based on 76 coded segments (segments per study: mean 4.75, SD 4.11; median 3.5, IQR 2.5-7). The frequently mentioned facilitators were the dimensions *fit into the workflow* and *evaluation of AI use*, with a strong connection between these dimensions (Figure 4C). The barriers centered on the dimension *reliability*, with a strong connection to the dimension *fit into the workflow* (Figure 4F).

### Reported Outcomes of AI Implementation

The included studies examined various outcomes stemming from the implementation of AI tools in medical imaging tasks. Of the 38 included studies, 31 (82%) reported efficiency outcomes, with 71% (22/31) of the studies showing enhanced efficiency, while 6% (2/31) of the studies reported a negative impact, and 23% (7/31) of the studies indicated no changes in efficiency. 13% (5/38) of the included studies assessed the impact of AI on workload or required work steps, with 80% (4/5) of the studies reporting reductions and 20% (1/5) of the studies indicating an increase. Of the 38 included studies, 16 (42%) reported on the performance of AI solutions in terms of changes in detection rates, need for human oversight, or quality of the AI-based results. In addition, 34% (13/38) discussed outcomes for patients, such as enhanced safety or quality control due to AI; a reduced time to diagnosis or treatment; prolonged stay in the emergency department; and increased detection rates, possibly leading to additional unnecessary treatments or increased workload [98]. The full details on the reported study outcomes are provided in Multimedia Appendix 7 [67-95,98,99, 101-104].

### Moderating Factors of AI Implementation

Of the 38 included studies, 18 (47%) identified moderators, which are defined as factors that influence AI use but are independent of the AI itself, such as the setting or the users. Details on the studies reporting moderators are provided in Multimedia Appendix 8 [69,70,75,77,78,80-82,84-86,91,93, 95,98,100,102,103].

The setting, precisely the shifts, times of the day, or whether it was a weekday or a weekend, was mentioned by 5% (2/38) of the studies [78,86]. Schmuelling et al [93] and Wong et al [102] also highlighted the significant influence of the clinical environment or pre-existing clinical workflows on AI implementation [93,102].

In addition, 21% (8/38) of the studies described that the implementation and use of AI are impacted by how health care professionals use the AI system, such as through personal preferences concerning their workflow or change in behaviors when they are not being observed. In total, 11% (4/38) examined the impact of human behavior on the evaluation of AI solutions in terms of interobserver variability or the missing reporting of errors.

In total, 26% (10/38) of the studies listed task-related factors, for example, differences due to input image quality, task type, or criticality of the findings. Moreover, 18% (7/38) of the studies noted that job experience or familiarity with AI has an impact on AI use.

Of the 38 included studies, 5 (13%) investigated physician performance when using AI regarding their job experience, with 20% (1/5) of the studies reporting no association [80]. Furthermore, 40% (2/5) of the studies reported a more positive AI use evaluation [69,84] or an enhanced detection rate [85] for less experienced readers, while 20% (1/5) of the studies reported that “the time to review the CAD images increased with the experience of the reader” [95].

### Additional Search to Include Recent Evidence

We searched 6 databases (PubMed, Web of Science, Embase, CENTRAL, Cochrane, and IEEE Xplore) to further identify recently published, relevant evidence, including review articles, in contrast to our original review process. While we retrieved and screened 1016 records, we identified 9 studies investigating facilitators and barriers of AI implementation in medical imaging. Among the 9 studies, 5 (56%) were scoping reviews, with 40% (2/5) of them focusing on AI implementation in health care in general [29,34], 40% (2/5) of the reviews studying AI for breast imaging [35,36], and 20% (1/5) of the reviews focusing on AI in radiology [37]. Only Chomutare et al [29] used a theoretical framework, the CFIR, to guide their analysis. All reviews provided a narrative synthesis of the results. In addition, of the 9 studies retrieved through the additional search, we identified 4 (44%) original studies, all using interviews as a qualitative methodology for studying facilitators and barriers of AI in medical imaging. Among those, 50% (2/4) of the studies did not study a specific AI implementation [38,39] and the other 50% (2/4) of the studies focused on specific AI solutions and were published after our second search [33,40]. Further details on these studies are provided in Multimedia Appendix 2.

## Discussion

### Principal Findings

Our systematic review provides, to the best of our knowledge, the first qualitative and quantitative synthesis that analyzes facilitators and barriers reported in studies on AI implementation in real-world clinical practice. Using our differentiation between the 3 levels of implementation, we were able to delve into the complexities of transferring AI technologies from model development and testing into the actual clinical environment [30]. To strengthen our conclusions, we used the SEIPS model, which is a strong asset for the system-based analysis of health care work environments [50]. In our analysis, we found that the frequency of various facilitators and barriers differed significantly across the stages of implementation. However, a consistently wide range of factors was identified, emphasizing the complex interplay of various elements when integrating AI into routine care processes. Consequently, our study offers a consolidated list of key factors that should be considered during AI implementation.

Focusing on categories across the implementation levels and matching them to work system elements can guide future implementation processes. In the conducted ENAs, the work system elements *tasks*, *tools and technology*, *organization*, and *people* were associated with the different axes, which provided a visualization of the importance of interactions between the work system elements. Missing in this categorization was the work system element *physical environment*, likely due to the diverse study settings and minimal impact of AI on work environments in the included studies. All studies focused on software as a medical device solutions that mostly did not alter their physical environment, and only 2 studies [89,104] reported physical changes because the AI solution was displayed on separate monitors. Referring to our resulting network graphs (Figure 4), it is noteworthy that the dimension *implementation procedure* was linked to work system elements *tasks* and *people*, while typically it is associated with organizational decisions [39,112]. Our classification showed that the included studies focused on evaluating AI on a microsystem level, that is, the individual health professionals and the tasks associated with AI use [113,114].

Studies describing external validations of AI solutions reported facilitators mostly related to the dimension *evaluation of AI use*, which was also the most prominent dimension overall. Barriers often stemmed from the AI technology itself, especially from the issues with *usability*. The focus of these networks highlights that external validation is still a part of the algorithm development process in which the clinical applicability of the AI solutions is being assessed. This is also supported by the outcomes reported in these studies, which were mostly time related, such as efficiency, treatment times, or workload. Moderating factors were not very prominent in these studies and were predominantly task related. These studies usually test the algorithm’s interaction with various work system elements for the first time under realistic conditions, which is often not done during the AI development phase before clinical validation [115].

Studies focusing on the initial implementation tested how AI solutions can be fitted into the existing workflow, while not yet being applied to all patients or cases. Barriers and facilitators in these studies mainly focus on the work system elements *people* and *tasks*, with most connections in the ENA stemming from this quadrant. In addition, these studies presented a broader spectrum of outcomes, such as satisfaction or patient outcomes. Moderating factors to AI use in these studies were also diverse, including experience of clinicians and their behavior. This focus aligns with the SEIPS model, which prioritizes the people and a human-centered design [19]. This resonates well with the identified initial implementation studies that tested and studied AI integration into the work system, and determined the necessary optimizations. The rising recognition of the significance of human-centered design and stakeholder engagement in the adoption of AI in health care is supported by our findings [14,35,116-118].

In the network analysis of studies assessing AI solutions that have been fully integrated into routine care, the dimension *fit into the workflow* emerges as the largest node of facilitators, with also the most connections, supporting the literature that highlights the integration of AI into work processes as crucial for success [10,12,109]. The themes we observed as being most important were *accessibility of results* and *no disruptions due to AI*, with the latter being mentioned positively by the absence of AI-related disruptions to the workflow. As workflow disruptions can increase the procedure duration, this is highly relevant in medical imaging, as radiologists and other physicians face increasing workloads and time pressures due to the large amount of medical imaging data to be interpreted [119,120]. Interestingly, barriers in these studies showed a strong connection between the dimensions *reliability* and *fit into the workflow*. This aligns with our recent findings that technical issues can largely impact the workflow, contrasting with the literature that often emphasizes ethical debates, medicolegal concerns, or AI explainability, which were less prominent in our analysis [112,121]. Nevertheless, most outcomes reported in these studies were positive, such as increased efficiency, improved detection rates, or reduced treatment times, potentially reflecting that only the AI solutions that have overcome most barriers manage the transfer from the initial development stage to full implementation [29].

### Comparison to Previous Work

Compared to previous research in the field, our results contribute important insights and show consistencies and discrepancies in AI implementation research. Few reviews have focused on the implementation of AI in clinical practice, and even fewer have specifically examined the facilitators and barriers to AI implementation. In our additional search, we only identified 5 scoping reviews targeting this topic in relation to AI for medical imaging. Hassan et al [34] provided a recent review on the facilitators and barriers to AI adoption, noting that most of the included studies focused on radiology and oncology. The authors identified 18 categories of facilitators and barriers, and similar to our findings, they observed that the same factor can be described as both a facilitator and a barrier [34]. However, because Hassan et al [34] do not offer a detailed overview of the included studies and only present a narrative synthesis, the comparison with our included studies, their settings, and designs is limited.

Lokaj et al [35] reviewed AI development and implementation for breast imaging diagnosis, identifying clinical workflow as a key facilitator. However, they emphasized technical aspects and algorithm development, with barriers such as data, evaluation, and validation issues. They noted the inclusion of very few prospective studies. In contrast, our review focuses on AI solutions evaluated after the development phase, in real-world clinical settings; therefore, technical aspects do not play a significant role in our developed set of facilitators and barriers.

Chomutare et al [29] also reviewed AI implementation in health care using the CFIR focusing on late-stage implementations. Despite including only 19 studies, they identified dimensions similar to ours, such as interoperability and transparency. Using ENAs based on implementation levels, our study provides a detailed overview of the facilitators and barriers at different implementation stages. Our findings further support the claim of Chomutare et al [29] that limited knowledge exists about the clinicians working with AI. Our review found that 29% (11/38) of the included studies incorporated user feedback, revealing a significant research gap. This underscores the need for research to adopt human-centered design, defined by the International Organization for Standardization standard 9241-210:2019 as follows: “an approach to interactive systems development that aims to make systems usable and useful by focusing on the users, their needs and requirements, and by applying human factors/ergonomics, and usability knowledge and techniques. This approach enhances effectiveness and efficiency, improves human well-being, user satisfaction, accessibility and sustainability; and counteracts possible adverse effects of use on human health, safety and performance” [122]. Using human-centered design principles is crucial for developing AI systems that benefit clinicians and patients [116,118].

Factors influencing AI adoption in health care are similar to those for other health information technologies, for example, electronic health records or e-prescription systems [123-125]. Key success factors, such as stakeholder involvement and system usability, are comparable across these technologies [126,127]. Recommendations for AI implementation can be drawn from health information technology research, such as that by Yen et al [128], who emphasize the importance of the sociotechnical context and longitudinal studies over cross-sectional outcomes. Although few of our included studies reported on the implementation process over time, our network analyses by implementation level can help identify the criteria that must be met in the course of AI tool transitions from research to clinical practice. AI introduces unique considerations to health care workflows, such as shared decision-making and human oversight [129], and presents new challenges requiring a broader understanding of the technology [130].

Clinicians need to understand the data used to train AI tools, as biases and limitations can arise, a point highlighted by Pierce et al [87] through their educational campaign before AI implementation. As AI solutions present the possibility of algorithmic bias, which might not be detected by clinicians, it is noteworthy that we identified user training and transparency as facilitators of AI implementation. The diverse nature of algorithmic biases, for example, stemming from biased training data, data gaps on underrepresented groups, human bias of the developers, or a lack of data standards, is an important information to be considered by the users [131-133]. Algorithmic bias holds the potential for patient harm, especially for populations considered disadvantaged [132]. While we identified strategies that can limit the impact of bias, such as user training, continuous monitoring, or transparency, most of the included studies did not explicitly mention bias, as described in by Wenderott et al [31]. Beyond algorithmic bias, it is also essential to address the legal and ethical challenges surrounding AI-supported decisions in health care [134]. Although these topics are widely discussed in research and politics, only 13% (5/38) of the studies we reviewed discussed medicolegal concerns in terms of data privacy concerns and legal implications. Thus, although AI solutions have been successfully implemented into routine medical care, issues of liability remain unresolved [135,136]. As AI continues to evolve and becomes more integrated into clinical practice, it is crucial to carefully consider these factors to ensure its safe, effective, and responsible use in health care settings.

### Limitations

Our study has a few limitations worth noting. First, we focused exclusively on AI tools in medical imaging, aiming to ensure the comparability of our findings. However, we encountered significant diversity in study settings, AI solutions, and purposes for decision-making or diagnostics. Because we only reviewed peer-reviewed original studies, some evaluations of AI implementation in health care might have been missed. Second, our findings showed more facilitators than barriers, which could be associated with a potential publication bias toward a more positive reporting of AI implementation, especially in combination with the high number of studies that reported a conflict of interest. In addition, we only searched for peer-reviewed literature, possibly missing reports on AI implementation from gray literature. AI implementation might also occur in clinical practice without scientific evaluation or reporting of results, which could also contribute to a publication bias. Third, the rapidly evolving nature of AI research indicates that certain processes or issues discussed in the studies may already be outdated by the time of publication, a challenge particularly relevant to the time-consuming process of systematic reviews, which often face delays from the literature search to final publication [32]. Therefore, while our review provides the first comprehensive, thorough, and methodologically rigorous overview of the facilitators and barriers to AI implementation in medical imaging, we recommend that future studies consider adopting shorter review cycles to ensure more timely publication and greater relevance in light of ongoing technical advancements. Fourth, facilitators and barriers were mainly extracted from study discussions, with separate reporting being rare, possibly introducing bias. In general, we noted that the descriptions of the implementation procedure and setting were sparse. Future research should provide details on their implementation strategy, processes, and subsequent adjustments to best integrate technology into the unique workflow [112]. This would enable comparisons across studies and facilitate learning in the scientific community. In addition, our established dimensions were formed inductively, requiring further validation. Fifth, while we used the SEIPS model for our analysis, we acknowledge that other frameworks exist such as the CFIR; the IDEAS framework; or the NASSS framework [22,24,26]. We planned to use the NASSS as specified in the review protocol but eventually chose the SEIPS model due to its human-centered and system-based approach [28]. Finally, our focus was on real-world investigations in clinical settings. Although our classification of “level of implementation” was useful for comparing different studies, its applicability to other clinical tasks, medical specialties, and work settings needs further examination. Furthermore, future studies should explore the impact of regulatory settings on research outcomes. While this was not feasible in our review due to the limited number of studies, the growing number of available AI algorithms and academic publications on AI in medicine will potentially provide sufficient data for these analyses [11,63].

### Conclusions

In conclusion, the facilitators and barriers identified in medical imaging studies have produced a comprehensive list of dimensions and themes essential for AI implementation in clinical care. Our research underscores the pressing necessity for holistic investigations into AI implementation, encompassing not only the technical aspects but also their impact on users, teams, and work processes. Furthermore, our results corroborate the future need for transparent reporting of AI implementation procedures. This transparency fosters knowledge exchange within the scientific community, facilitating the translation of research findings into actionable strategies for clinical care. A deeper understanding of how AI solutions affect clinicians and their workflows can help reduce clinician workload and improve patient care.

## Appendix

Multimedia Appendix 1 PRISMA (Preferred Reporting Items for Systematic Reviews and Meta-Analyses) checklist.

Multimedia Appendix 2 Additional search.

Multimedia Appendix 3 Geographical comparison.

Multimedia Appendix 4 Artificial intelligence solutions.

Multimedia Appendix 5 Overview on surveys used in the included publications.

Multimedia Appendix 6 Details on the extracted themes.

Multimedia Appendix 7 Outcomes extracted from the included publications.

Multimedia Appendix 8 Moderators extracted from the included publications.

Multimedia Appendix 9 ChatGPT transcript.

## Abbreviations

AI: artificial intelligence
CFIR: Consolidated Framework for Implementation Research
ENA: epistemic network analysis
IDEAS: integrate, design, assess, and share
NASSS: nonadoption, abandonment, scale-up, spread, and sustainability
PICO: population, intervention, comparison, and outcome
PRISMA: Preferred Reporting Items for Systematic Reviews and Meta-Analyses
SEIPS: Systems Engineering Initiative for Patient Safety

## Data Availability

The datasets generated or analyzed during this study are available from the corresponding author on reasonable request.
