# Supplementary material for: Facilitators and Barriers to Implementing AI in Routine Medical Imaging: Systematic Review and Qualitative Analysis
Source: J Med Internet Res. 2025 Jul 21;27:e63649. doi: 10.2196/63649 (PMC12322614; doi:10.2196/63649)
Supplement: Multimedia Appendix 2 [file jmir_v27i1e63649_app2.docx]

### **Multimedia Appendix 2. Additional search.**

The process of conducting a systematic review typically takes several months to a year, as it involves thorough data screening and analysis. As a result, by the time the review is published, new evidence may already be available. To ensure we provide the most up-to-date information, we conducted a rapid review on November 28, 2024, focusing specifically on the facilitators and barriers to AI implementation.

We used the following search term: “facilitator* AND barrier* AND (artificial intelligence OR ai OR machine learning)“.

One reviewer (KW) screened all title and abstracts and one reviewer from the team (JK) screened 20% (203 records). Both reviewers screened all included full-texts. The agreement between both reviewers was 100%. See Figure 1 for the flowchart of our additional search. Table 1 and 2 list the eligible studies we identified in our additional search.

## Flowchart


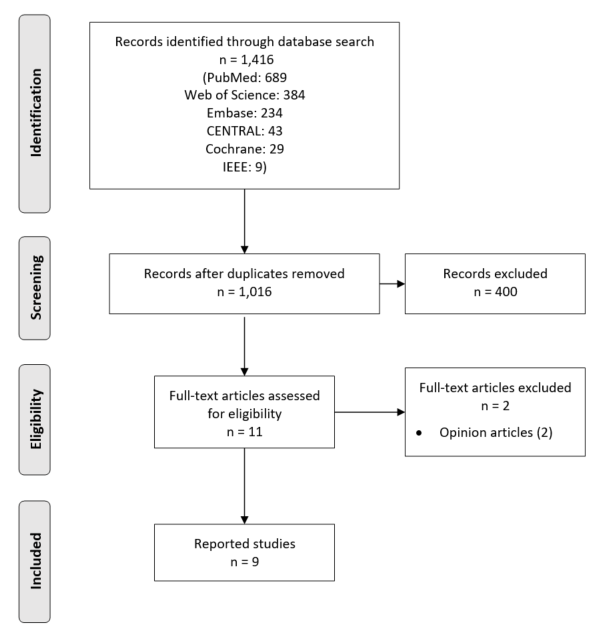


Figure 1 Flowchart of the additional search

## Results

### Reviews:

Table 1 Reviews retrieved from the additional search

| **Author(s)** | **Year** | **Type** | **Dates** | **Databases** | **Screening** | **Included** | **Framework** | **Focus** |
| --- | --- | --- | --- | --- | --- | --- | --- | --- |
| Chomutare et al. [1] | 2022 | Scoping Review | 2015-2021 | Pubmed, IEEE, ACM, Google Scholar, Web of Science | 2784 | 19 | CFIR | AI in healthcare |
| Eltawil el al. [2] | 2023 | Scoping Review | 2000-2023 | Ovid Medline, Embase | 513 | 12 | NA | Radiology |
| Hassan et al. [3] | 2024 | Scoping Review | 2011-2023 | MEDLINE,IEEE Xplore, sciencedirect database | 2514 | 50 | NA | AI adoption in healthcare |
| Lokaj et al. [4] | 2023 | Scoping Review | 2012-2023 | Pubmed, Web of Science, CINHAL, Embase, IEEE, arxiv | 1476 | 107 | NA | AI in breast imaging |
| Masud et al. [5] | 2019 | Scoping Review | 2010-2019 | MEDLINE | 523 | 9 | NA | Computer-Aided Detection for Breast Cancer Screening |

Abbreviations: NA not applicable, CFIR Consolidated Framework for Implementation Research

### Original Research:

Table 2 Original studies retrieved from the additional search

| **Author(s)** | **Year** | **Country** | **Study type** | **Participants** | **Framework** | **Focus** |
| --- | --- | --- | --- | --- | --- | --- |
| Liao et al. [6] | 2024 | China | Qualitative interview study | 33 stakeholders from healthcare, 7 vendors of AI software | CFIR | Imaging-based diagnostic AI-assisted decision-making software |
| Strohm et al. [7] | 2020 | Netherlands | Qualitative Interview Study | 24 interviews | NASSS | AI in radiology |
| Swillens et al. [8] | 2023 | Netherlands | Narrative literature review, eSurvey, Interviews | 70 eSurvey respondents, 16 interviews | NA | Computational pathology adoption in oncological pathology |
| Wenderott et al. [9] | 2024 | Germany | Before-After Interview Study | 9 radiologists, 19 interviews | SEIPS | CDSS for prostate MRI |

Abbreviations: NA not applicable, CFIR Consolidated Framework for Implementation Research, NASSS Non-adoption, Abandonment, Scale-up, Spread, and Sustainability framework, SEIPS Systems Engineering Initiative for Patient Safety model

## References

1. Chomutare T, Tejedor M, Svenning TO, Marco-Ruiz L, Tayefi M, Lind K, Godtliebsen F, Moen A, Ismail L, Makhlysheva A, Ngo PD. Artificial Intelligence Implementation in Healthcare: A Theory-Based Scoping Review of Barriers and Facilitators. Int J Environ Res Public Health 2022;19(23):16359. doi: 10.3390/ijerph192316359

2. Eltawil FA, Atalla M, Boulos E, Amirabadi A, Tyrrell PN. Analyzing Barriers and Enablers for the Acceptance of Artificial Intelligence Innovations into Radiology Practice: A Scoping Review. Tomography 2023 Jul 28;9(4):1443–1455. doi: 10.3390/tomography9040115

3. Hassan M, Kushniruk A, Borycki E. Barriers to and Facilitators of Artificial Intelligence Adoption in Health Care: Scoping Review. JMIR Hum Factors 2024 Aug 29;11:e48633. doi: 10.2196/48633

4. Lokaj B, Pugliese M-T, Kinkel K, Lovis C, Schmid J. Barriers and Facilitators of Artificial Intelligence Conception and Implementation for Breast Imaging Diagnosis in Clinical Practice: A Scoping Review. Eur Radiol 2023;34(3):2096–2109. doi: 10.1007/s00330-023-10181-6

5. Masud R, Al-Rei M, Lokker C. Computer-Aided Detection for Breast Cancer Screening in Clinical Settings: Scoping Review. JMIR Med Inform 2019 Jul 18;7(3):e12660. doi: 10.2196/12660

6. Liao X, Yao C, Jin F, Zhang J, Liu L. Barriers and Facilitators to Implementing Imaging-Based Diagnostic Artificial Intelligence-Assisted Decision-Making Software in Hospitals in China: A Qualitative Study Using the Updated Consolidated Framework for Implementation Research. BMJ Open 2024 Sep;14(9):e084398. doi: 10.1136/bmjopen-2024-084398

7. Strohm L, Hehakaya C, Ranschaert ER, Boon WPC, Moors EHM. Implementation of Artificial Intelligence (AI) Applications in Radiology: Hindering and Facilitating Factors. Eur Radiol 2020;30(10):5525–5532. doi: 10.1007/s00330-020-06946-y

8. Swillens JEM, Nagtegaal ID, Engels S, Lugli A, Hermens RPMG, Van Der Laak JAWM. Pathologists’ first opinions on barriers and facilitators of computational pathology adoption in oncological pathology: an international study. Oncogene 2023 Sep 15;42(38):2816–2827. doi: 10.1038/s41388-023-02797-1

9. Wenderott K, Krups J, Luetkens JA, Gambashidze N, Weigl M. Prospective Effects of an Artificial Intelligence-Based Computer-Aided Detection System for Prostate Imaging on Routine Workflow and Radiologists’ Outcomes. Eur J Radiol 2024 Jan;170:111252. doi: 10.1016/j.ejrad.2023.111252
