# Supplementary material for: Facilitators and Barriers to Implementing AI in Routine Medical Imaging: Systematic Review and Qualitative Analysis
Source: J Med Internet Res. 2025 Jul 21;27:e63649. doi: 10.2196/63649 (PMC12322614; doi:10.2196/63649)
Supplement: Multimedia Appendix 3 [file jmir_v27i1e63649_app3.docx]

### **Multimedia Appendix 3. Geographical comparison.**

Regulatory frameworks for AI in healthcare differ between countries or regions of the world. While our review does not include a sufficient number of studies for an in-depth geographical comparison and analysis, here we provide an initial comparison of the extracted qualitative data between the two main geographical clusters, the EU and the US, which are commonly contrasted when comparing the regulatory landscape of AI in healthcare [1–3]. We did not observe any striking differences, except for in the US more full implementation studies were conducted, potentially showing that already more AI solutions are integrated into clinical practice. As this is only a first overview and initial interpretation this should be explored in further research.

Table 2 Comparison of dimensions between studies conducted in the US vs. EU

| **Dimension** | **US (16 studies)** | **EU (10 studies)** |
| --- | --- | --- |
| AI Impact on Clinicians Role | 25% | 0% |
| Attitudes & Values | 19% | 0% |
| Evaluation of AI use | 56% | 50% |
| Explainability of AI | 13% | 40% |
| Fit into the workflow | 63% | 50% |
| Implementation procedure | 44% | 0% |
| Individual Work organization | 19% | 0% |
| Interoperability | 6% | 40% |
| Medicolegal Concerns | 0% | 20% |
| Reliability | 19% | 40% |
| Stakeholder involvement | 31% | 10% |
| Usability | 38% | 0% |

Table 3 Comparison of the level of implementation of studies between the US and EU

| **Level of Implementation** | **US (16 studies)** | **EU (10 studies)** |
| --- | --- | --- |
| External | 13% | 40% |
| Initial | 38% | 20% |
| Full | 50% | 40% |

**References**

1. Vokinger KN, Gasser U. Regulating AI in medicine in the United States and Europe. Nat Mach Intell 2021 Sep 10;3(9):738–739. doi: 10.1038/s42256-021-00386-z

2. Muehlematter UJ, Daniore P, Vokinger KN. Approval of Artificial Intelligence and Machine Learning-Based Medical Devices in the USA and Europe (2015–20): A Comparative Analysis. Lancet Digit Health Elsevier; 2021;3(3):e195–e203. PMID:33478929

3. Romagnoli A, Ferrara F, Langella R, Zovi A. Healthcare Systems and Artificial Intelligence: Focus on Challenges and the International Regulatory Framework. Pharm Res 2024 Apr;41(4):721–730. doi: 10.1007/s11095-024-03685-3
