# Supplementary material for: Facilitators and Barriers to Implementing AI in Routine Medical Imaging: Systematic Review and Qualitative Analysis
Source: J Med Internet Res. 2025 Jul 21;27:e63649. doi: 10.2196/63649 (PMC12322614; doi:10.2196/63649)
Supplement: Multimedia Appendix 4 [file jmir_v27i1e63649_app4.docx]

### **Multimedia Appendix 4. Artificial intelligence solutions.**

The included studies encompass a variety of AI solutions; therefore Table 3 presents the source of the AI solutions used and the purpose of AI use. More details can be found in Wenderott et al. (2024) Effects of artificial intelligence implementation on efficiency in medical imaging - A systematic literature review and meta-analysis*. npj Digital Medicine.* doi: 10.1038/s41746-024-01248-9 [1]

Table 4 Details on AI solutions used in the included studies

| **Study** | **Source of AI** | **Purpose of AI use** |
| --- | --- | --- |
| Arbabshirani et al. [2] | Developed by the authors | Detection of ICH + reprioritization of worklist |
| Batra et al. [3] | Aidoc Medical, Tel Aviv, Israel | Detection of PE + reprioritization of worklist |
| Carlile et al. [4] | Department of radiology at UC San Diego Health | Detection of COVID-19 |
| Cha et al. [5] | Elguindi et al. [6] | Contouring of organs at risk for radiotherapy planning |
| Cheikh et al. [7] | Aidoc Medical, Tel Aviv, Israel | Detection of PE |
| Chen et al. [8] | Developed by the authors | Lesion detection and lesion scoring (ASPECTS score) |
| Conant et al. [9] | iCAD, Nashua, NH, USA | Breast cancer detection |
| Davis et al. [10] | Aidoc Medical, Tel Aviv, Israel | Detection of ICH + flagging of positive cases |
| Diao et al. [11] | Infervision Technology Co., Ltd., Beijing, China | Detection of lung nodules on CT scans |
| Duron et al. [12] | Gleamer, Paris, France | Detection of fractures |
| Elijovich et al. [13] | Viz.ai, San Francisco, CA, USA | Detection of LVO + sending alert to treating clinician |
| Ginat [14] | Aidoc Medical, Tel Aviv, Israel | Detection of ICH + reprioritization of worklist |
| Hassan et al. [15] | Viz.ai, San Francisco, CA, USA | Detection of LVO + sending alert to treating clinician |
| Jones et al. [16] | Annalise-AI, Sydney, Australia | Analysis of radiographs |
| Ladabaum et al. [17] | Medtronic, Minneapolis, MN, USA | Detection of adenomas during colonoscopy |
| Levy et al. [18] | Medtronic, Minneapolis, MN, USA | Detection of adenomas during colonoscopy |
| Marwaha et al. [19] | FDNA Inc., Sunrise, FL, USA | Detection of rare genetic diseases on face photographs |
| Mueller et al. [20] | Siemens Healthineers, Erlangen, Germany | Analysis of chest CT scans |
| Nehme et al. [21] | Medtronic, Minneapolis, MN, USA | Detection of adenomas during colonoscopy |
| Oppenheimer et al. [22] | Gleamer, Paris, France | Detection of fractures |
| Pierce et al. [23] | NI | Detection of pneumothorax + alert or prioritization |
| Potrezke et al. [24] | Developed by the authors | Kidney volume determination |
| Quan et al. [25] | EndoVigilant Inc., Maryland, USA | Detection of adenomas during colonoscopy |
| Raya-Povedano et al. [26] | ScreenPoint Medical, Nijmegen, The Netherlands | Breast cancer detection + exclusion of low suspicious cases |
| Ruamviboonsuk et al. [27] | Gulshan et al. [28] | Detection of DR |
| Sandbank et al. [29] | Developed by the authors | Breast biopsy grading + cancer detection |
| Schmuelling et al. [30] | Aidoc Medical, Tel Aviv, Israel | Detection of PE + reprioritization of worklist |
| Seyam et al. [31] | Aidoc Medical, Tel Aviv, Israel | Detection of ICH + alert in case of a positive finding |
| Tchou et al. [32] | Hologic, Marlborough, MA, USA | Breast cancer detection |
| Tricarico et al. [33] | AITEM Solutions, Turin, Italy | Detection of COVID-19 + prioritization of urgent cases |
| Vassallo et al. [34] | Retico et al. [35] | Detection of lung nodules |
| Wang et al. [36] | Shanghai Wision AI Co., Ltd. | Detection of adenomas during colonoscopy |
| Wang et al. [37] | Developed by the authors | Detection of COVID-19 + flagging of positive cases |
| Wittenberg et al. [38] | Philipps Healthcare, Best, The Netherlands | Detection of PE |
| Wong et al. [39] | Limbus AI, Regina, Saskatchewan, Canada | Contouring of organs at risk for radiotherapy planning |
| Wong et al. [40] | Brown et al. [41] | Assistance in checking endotracheal tube placement |
| Yang et al. [42] | Shenzen SiBright Co. Ltd., China | Detection of DR |
| Zia et al. [43] | Aidoc Medical, Tel Aviv, Israel | Detection of ICH + alert in case of a positive finding |
| **Notes:** Abbreviations: ASPECTS Alberta Stroke Program Early Computed Tomography Score; CT computed tomography: DR diabetic retinopathy; ICH intracranial haemorrhage; LVO large vessel occlusion; NI no information; PE pulmonary embolism | | |

**References**

1. Wenderott K, Krups J, Zaruchas F, Weigl M. Effects of Artificial Intelligence Implementation on Efficiency in Medical Imaging—a Systematic Literature Review and Meta-Analysis. Npj Digit Med 2024 Sep 30;7(1):265. doi: 10.1038/s41746-024-01248-9

2. Arbabshirani MR, Fornwalt BK, Mongelluzzo GJ, Suever JD, Geise BD, Patel AA, Brandon K.; ORCID: https://orcid.org/0000-0002-6231-9442 MGJA-F. Advanced Machine Learning in Action: Identification of Intracranial Hemorrhage on Computed Tomography Scans of the Head with Clinical Workflow Integration. Npj Digit Med 2018;1(1):9. doi: 10.1038/s41746-017-0015-z

3. Batra K, Xi Y, Bhagwat S, Espino A, Peshock R. Radiologist Worklist Reprioritization Using Artificial Intelligence: Impact on Report Turnaround Times for CTPA Examinations Positive for Acute Pulmonary Embolism. AJR Am J Roentgenol 2023 Apr; doi: 10.2214/AJR.22.28949

4. Carlile M, Hurt B, Hsiao A, Hogarth M, Longhurst CA, Dameff C. Deployment of Artificial Intelligence for Radiographic Diagnosis of Covid‐19 Pneumonia in the Emergency Department. J Am Coll Emerg Physicians Open 2020 Dec;1(6):1459–1464. doi: 10.1002/emp2.12297

5. Cha E, Elguindi S, Onochie I, Gorovets D, Deasy JO, Zelefsky M, Gillespie EF. Clinical Implementation of Deep Learning Contour Autosegmentation for Prostate Radiotherapy. Radiother Oncol 2021;159:1–7. doi: 10.1016/j.radonc.2021.02.040

6. Elguindi S, Zelefsky MJ, Jiang J, Veeraraghavan H, Deasy JO, Hunt MA, Tyagi N. Deep learning-based auto-segmentation of targets and organs-at-risk for magnetic resonance imaging only planning of prostate radiotherapy. Phys Imaging Radiat Oncol 2019 Oct;12:80–86. doi: 10.1016/j.phro.2019.11.006

7. Cheikh AB, Gorincour G, Nivet H, May J, Seux M, Calame P, Thomson V, Delabrousse E, Crombé A. How Artificial Intelligence Improves Radiological Interpretation in Suspected Pulmonary Embolism. Eur Radiol 2022 Mar 22;32(9):5831–5842. doi: 10.1007/s00330-022-08645-2

8. Chen W, Wu J, Wei R, Wu S, Xia C, Wang D, Liu D, Zheng L, Zou T, Li R, Qi X, Zhang X. Improving the Diagnosis of Acute Ischemic Stroke on Non-Contrast Ct Using Deep Learning: A Multicenter Study. Insights Imaging 2022 Dec;13(1):184. doi: 10.1186/s13244-022-01331-3

9. Conant EF, Toledano AY, Periaswamy S, Fotin SV, Go J, Boatsman JE, Hoffmeister JW. Improving Accuracy and Efficiency with Concurrent Use of Artificial Intelligence for Digital Breast Tomosynthesis. Radiol Artif Intell 2019 Jul;1(4):e180096. doi: 10.1148/ryai.2019180096

10. Davis MA, Rao B, Cedeno PA, Saha A, Zohrabian VM. Machine Learning and Improved Quality Metrics in Acute Intracranial Hemorrhage by Noncontrast Computed Tomography. Curr Probl Diagn Radiol 2022 Jul;51(4):556–561. doi: 10.1067/j.cpradiol.2020.10.007

11. Diao K, Chen Y, Liu Y, Chen B, Li W, Zhang L, YL Q, Zhang T, Zhang Y, Wu M, Li K, Song B. Diagnostic Study on Clinical Feasibility of an AI-Based Diagnostic System as a Second Reader on Mobile CT Images: A Preliminary Result. Ann Transl Med 2022 Jun;10(12):668. doi: 10.21037/atm-22-2157

12. Duron L, Ducarouge A, Gillibert A, Lainé J, Allouche C, Cherel N, Zhang Z, Nitche N, Lacave E, Pourchot A, Felter A, Lassalle L, Regnard N-E, Feydy A. Assessment of an AI Aid in Detection of Adult Appendicular Skeletal Fractures by Emergency Physicians and Radiologists: A Multicenter Cross-sectional Diagnostic Study. Radiology 2021 Jul;300(1):120–129. doi: 10.1148/radiol.2021203886

13. Elijovich L, Dornbos III D, Nickele C, Alexandrov A, Inoa-Acosta V, Arthur AS, Hoit D. Automated Emergent Large Vessel Occlusion Detection by Artificial Intelligence Improves Stroke Workflow in a Hub and Spoke Stroke System of Care. J NeuroInterventional Surg 2022 Jul;14(7):704–708. doi: 10.1136/neurintsurg-2021-017714

14. Ginat D. Implementation of Machine Learning Software on the Radiology Worklist Decreases Scan View Delay for the Detection of Intracranial Hemorrhage on CT. Brain Sci D. Ginat, Department of Radiology, University of Chicago, Chicago, IL 60615, United States. E-mail: dginat@radiology.bsd.uchicago.edu Switzerland: MDPI; 2021;11(7):832. doi: 10.3390/brainsci11070832

15. Hassan A, Ringheanu V, Tekle W. The Implementation of Artificial Intelligence Significantly Reduces Door-in-Door-Out Times in a Primary Care Center Prior to Transfer. Interv Neuroradiol J Peritherapeutic Neuroradiol Surg Proced Relat Neurosci 2022 Aug;15910199221122848. doi: 10.1177/15910199221122848

16. Jones CM, Danaher L, Milne MR, Tang C, Seah J, Oakden-Rayner L, Johnson A, Buchlak QD, Esmaili N. Assessment of the Effect of a Comprehensive Chest Radiograph Deep Learning Model on Radiologist Reports and Patient Outcomes: A Real-World Observational Study. BMJ Open 2021 Dec;11(12):e052902. doi: 10.1136/bmjopen-2021-052902

17. Ladabaum U, Shepard J, Weng Y, Desai M, Singer SJ, Mannalithara A. Computer-Aided Detection of Polyps Does Not Improve Colonoscopist Performance in a Pragmatic Implementation Trial. Gastroenterology 2023 Mar;164(3). doi: 10.1053/j.gastro.2022.12.004

18. Levy I, Bruckmayer L, Klang E, Ben-Horin S, Kopylov U. Artificial Intelligence-Aided Colonoscopy Does Not Increase Adenoma Detection Rate in Routine Clinical Practice. Am J Gastroenterol 2022 Nov;117(11):1871–1873. doi: 10.14309/ajg.0000000000001970

19. Marwaha A, Chitayat D, Meyn M, Mendoza-Londono R, Chad L. The Point-of-Care Use of a Facial Phenotyping Tool in the Genetics Clinic: Enhancing Diagnosis and Education with Machine Learning. Am J Med Genet A 2021 Apr;185(4):1151–1158. doi: 10.1002/ajmg.a.62092

20. Mueller FC, Raaschou H, Akhtar N, Brejnebol M, Collatz L, Andersen MB. Impact of Concurrent Use of Artificial Intelligence Tools on Radiologists Reading Time: A Prospective Feasibility Study. Acad Radiol 2022;29(7):1085–1090. doi: 10.1016/j.acra.2021.10.008

21. Nehme F, Coronel E, Barringer D, Romero L, Shafi M, Ross W, Ge P. Performance and Attitudes Toward Real-time Computer-aided Polyp Detection during Colonoscopy in a Large Tertiary Referral Center in the United States. Gastrointest Endosc 2023 Feb; doi: 10.1016/j.gie.2023.02.016

22. Oppenheimer J, Lüken S, Hamm B, Niehues S. A Prospective Approach to Integration of AI Fracture Detection Software in Radiographs into Clinical Workflow. Life Basel Switz 2023 Jan;13(1). doi: 10.3390/life13010223

23. Pierce J, Rosipko B, Youngblood L, Gilkeson R, Gupta A, Bittencourt L. Seamless Integration of Artificial Intelligence Into the Clinical Environment: Our Experience With a Novel Pneumothorax Detection Artificial Intelligence Algorithm. J Am Coll Radiol JACR 2021 Nov;18(11):1497–1505.

24. Potretzke T, Korfiatis P, Blezek D, Edwards M, Klug J, Cook C, Gregory A, Harris P, Chebib F, Hogan M, Torres V, Bolan C, Sandrasegaran K, Kawashima A, Collins J, Takahashi N, Hartman R, Williamson E, King B, Callstrom M, Erickson B, Kline T. Clinical Implementation of an Artificial Intelligence Algorithm for Magnetic Resonance-Derived Measurement of Total Kidney Volume. Mayo Clin Proc 2023 May;98(5):689–700. doi: 10.1016/j.mayocp.2022.12.019

25. Quan SY, Wei MT, Lee J, Mohi-Ud-Din R, Mostaghim R, Sachdev R, Siegel D, Friedlander Y, Friedland S. Clinical Evaluation of a Real-Time Artificial Intelligence-Based Polyp Detection System: A US Multi-Center Pilot Study. Sci Rep 2022 Apr 21;12(1):6598. doi: 10.1038/s41598-022-10597-y

26. Raya-Povedano JL, Romero-Martín S, Elías-Cabot E, Gubern-Mérida A, Rodríguez-Ruiz A, Álvarez-Benito M. AI-based Strategies to Reduce Workload in Breast Cancer Screening with Mammography and Tomosynthesis: A Retrospective Evaluation. Radiology 2021 Jul;300(1):57–65. doi: 10.1148/radiol.2021203555

27. Ruamviboonsuk P, Tiwari R, Sayres R, Nganthavee V, Hemarat K, Kongprayoon A, Raman R, Levinstein B, Liu Y, Schaekermann M, Lee R, Virmani S, Widner K, Chambers J, Hersch F, Peng L, Webster DR. Real-Time Diabetic Retinopathy Screening by Deep Learning in a Multisite National Screening Programme: A Prospective Interventional Cohort Study. Lancet Digit Health 2022 Apr;4(4). doi: 10.1016/S2589-7500(22)00017-6

28. Gulshan V, Peng L, Coram M, Stumpe MC, Wu D, Narayanaswamy A, Venugopalan S, Widner K, Madams T, Cuadros J, Kim R, Raman R, Nelson PC, Mega JL, Webster DR. Development and Validation of a Deep Learning Algorithm for Detection of Diabetic Retinopathy in Retinal Fundus Photographs. JAMA 2016 Dec 13;316(22):2402. doi: 10.1001/jama.2016.17216

29. Sandbank J, Bataillon G, Nudelman A, Krasnitsky I, Mikulinsky R, Bien L, Thibault L, Albrecht Shach A, Sebag G, Clark D, Laifenfeld D, Schnitt S, Linhart C, Vecsler M, Vincent-Salomon A. Validation and Real-World Clinical Application of an Artificial Intelligence Algorithm for Breast Cancer Detection in Biopsies. Npj Breast Cancer 2022 Dec;8(1):129. doi: 10.1038/s41523-022-00496-w

30. Schmuelling L, Franzeck FC, Nickel CH, Mansella G, Bingisser R, Schmidt N, Stieltjes B, Bremerich J, Sauter AW, Weikert T, Sommer G. Deep Learning-Based Automated Detection of Pulmonary Embolism on CT Pulmonary Angiograms: No Significant Effects on Report Communication Times and Patient Turnaround in the Emergency Department Nine Months After Technical Implementation. Eur J Radiol 2021 Aug;141:109816. doi: 10.1016/j.ejrad.2021.109816

31. Seyam M, Weikert T, Sauter A, Brehm A, Psychogios M-N, Blackham KA. Utilization of Artificial Intelligence-based Intracranial Hemorrhage Detection on Emergent Noncontrast CT Images in Clinical Workflow. Radiol Artif Intell 2022;4(2):e210168.

32. Tchou PM, Haygood TM, Atkinson EN, Stephens TW, Davis PL, Arribas EM, Geiser WR, Whitman GJ. Interpretation Time of Computer-aided Detection at Screening Mammography. Radiology 2010 Oct;257(1):40–46. doi: 10.1148/radiol.10092170

33. Tricarico D, Calandri M, Barba M, Piatti C, Geninatti C, Basile D, Gatti M, Melis M, Veltri A. Convolutional Neural Network-Based Automatic Analysis of Chest Radiographs for the Detection of COVID-19 Pneumonia: A Prioritizing Tool in the Emergency Department, Phase I Study and Preliminary “Real Life” Results. Diagnostics 2022;12(3):570. doi: https://dx.doi.org/10.3390/diagnostics12030570 PT - Article

34. Vassallo L, Traverso A, Agnello M, Bracco C, Campanella D, Chiara G, Fantacci ME, Lopez Torres E, Manca A, Saletta M, Giannini V, Mazzetti S, Stasi M, Cerello P, Regge D. A Cloud-Based Computer-Aided Detection System Improves Identification of Lung Nodules on Computed Tomography Scans of Patients with Extra-Thoracic Malignancies. Eur Radiol 2019 Jan;29(1):144–152. doi: 10.1007/s00330-018-5528-6

35. Retico A, Delogu P, Fantacci ME, Gori I, Preite Martinez A. Lung nodule detection in low-dose and thin-slice computed tomography. Comput Biol Med 2008 Apr;38(4):525–534. doi: 10.1016/j.compbiomed.2008.02.001

36. Wang P, Berzin TM, Glissen Brown JR, Bharadwaj S, Becq A, Xiao X, Liu P, Li L, Song Y, Zhang D, Li Y, Xu G, Tu M, Liu X. Real-Time Automatic Detection System Increases Colonoscopic Polyp and Adenoma Detection Rates: A Prospective Randomised Controlled Study. Gut 2019 Oct;68(10):1813–1819. doi: 10.1136/gutjnl-2018-317500

37. Wang M, Xia C, Huang L, Xu S, Qin C, Liu J, Cao Y, Yu P, Zhu T, Zhu H, Wu C, Zhang R, Chen X, Wang J, Du G, Zhang C, Wang S, Chen K, Liu Z, Xia L, Wang W. Deep Learning-Based Triage and Analysis of Lesion Burden for Covid-19: A Retrospective Study with External Validation. Lancet Digit Health 2020 Oct;2(10):e506–e515. doi: 10.1016/S2589-7500(20)30199-0

38. Wittenberg R, Berger FH, Peters JF, Weber M, van Hoorn F, Beenen LFM, van Doorn MMAC, van Schuppen J, Zijlstra IjAJ, Prokop M, Schaefer-Prokop CM. Acute Pulmonary Embolism: Effect of a Computer-assisted Detection Prototype on Diagnosis—An Observer Study. Radiology 2012 Jan;262(1):305–313. doi: 10.1148/radiol.11110372

39. Wong J, Huang V, Wells D, Giambattista J, Giambattista J, Kolbeck C, Otto K, Saibishkumar EP, Alexander A. Implementation of Deep Learning-Based Auto-Segmentation for Radiotherapy Planning Structures: A Workflow Study at Two Cancer Centers. Radiat Oncol 2021 Dec;16(1):101. doi: 10.1186/s13014-021-01831-4

40. Wong K, Homer S, Wei S, Yaghmai N, Estrada Paz O, Young T, Buhr R, Barjaktarevic I, Shrestha L, Daly M, Goldin J, Enzmann D, Brown M. Integration and Evaluation of Chest X-Ray Artificial Intelligence in Clinical Practice. J Med Imaging 2023 Sep;10(5):051805. doi: 10.1117/1.JMI.10.5.051805

41. Brown MS, Wong K-P, Shrestha L, Wahi-Anwar M, Daly M, Foster G, Abtin F, Ruchalski KL, Goldin JG, Enzmann D. Automated Endotracheal Tube Placement Check Using Semantically Embedded Deep Neural Networks. Acad Radiol 2023 Mar;30(3):412–420. doi: 10.1016/j.acra.2022.04.022

42. Yang Y, Pan J, Yuan M, Lai K, Xie H, Ma L, Xu S, Deng R, Zhao M, Luo Y, Lin X. Performance of the AIDRScreening System in Detecting Diabetic Retinopathy in the Fundus Photographs of Chinese Patients: A Prospective, Multicenter, Clinical Study. Ann Transl Med 2022 Oct;10(20):1088. doi: 10.21037/atm-22-350

43. Zia A, Fletcher C, Bigwood S, Ratnakanthan P, Seah J, Lee R, Kavnoudias H, Law M. Retrospective Analysis and Prospective Validation of an Ai-Based Software for Intracranial Haemorrhage Detection at a High-Volume Trauma Centre. Sci Rep 2022 Nov;12(1):19885. doi: 10.1038/s41598-022-24504-y
