# Supplementary material for: Facilitators and Barriers to Implementing AI in Routine Medical Imaging: Systematic Review and Qualitative Analysis
Source: J Med Internet Res. 2025 Jul 21;27:e63649. doi: 10.2196/63649 (PMC12322614; doi:10.2196/63649)
Supplement: Multimedia Appendix 5 [file jmir_v27i1e63649_app5.docx]

### **Multimedia Appendix 5. Overview on surveys used in the included publications.**

As indicated in Table 2 in the main text, some of the studies used surveys to assess additional outcomes. Details on the survey method and the respective population are found in Table 4. We grouped the question the authors asked according to our dimensions.

Table 5 Surveys used in the included studies

| **Study** | **Population** | **Method** | **Dimensions** | **Questions** |
| --- | --- | --- | --- | --- |
| Carlile et al. [1] | 45 radiologists (24 attendings, 21 residents) | 3-point survey | Evaluation of AI Use | Impact of AI on decision-making  Contribution of AI to decision-making |
|  |  |  | Usability | Ease of use |
| Cheikh et al. [2] | 79 radiologists | 5-point survey | Evaluation of AI Use | Impact of AI on diagnostic confidence  Impact of AI on reading time |
| Diao et al. [3] | 7 radiologists | Questionnaire | Reliability | AI performance |
| Jones et al. [4] | 10 radiologists | 5-point survey | Attitudes & Values | Change in attitude through AI use |
|  |  |  | Evaluation of AI use | Impact of AI on reading time  Satisfaction with AI |
|  |  |  | Usability | System Usability Scale |
| Marwaha et al. [5] | 10 provider responses | 5-point survey | Attitudes & Values | Openness to AI |
|  |  |  | Evaluation of AI Use | Usefulness of AI |
|  |  |  | Medicolegal Concerns | Concerns of patients |
|  |  |  | Reliability | Accuracy of AI |
|  |  |  | Usability | Ease of use |
| Mueller et al. [6] | 2 radiologists | Questionnaire | Evaluation of AI Use | Additional information through use of AI  Impact of AI on case overview  Impact of AI on diagnostic confidence  Impact of AI on diagnosis  Impact of AI on reading time |
| Nehme et al. [7] | 16 physicians,  16 staff members | 10-question survey prior to AI implementation | Attitudes & Values | Attitude towards AI  Enthusiasm for GI Genius  Experience with AI |
|  |  |  | Evaluation of AI Use | Expectations: Impact on diagnosis  Expectations: Impact on procedure time |
|  |  |  | Medicolegal Concerns | Concerns regarding AI |

|  | 17 physicians,  13 staff members | 10-question survey after AI implementation | Attitudes & Values | Change in attitude through AI use  Continuation of AI use |
| --- | --- | --- | --- | --- |

|  |  |  | Evaluation of AI Use | Impact of AI on diagnosis  Impact of AI on procedure time |
| --- | --- | --- | --- | --- |
|  |  |  | Fit into the Workflow | Distraction due to AI |
|  |  |  | Individual Work Organization | Use of AI |
| Ruamviboonsuk et al. [8] | 12 staff members | 5-point survey | Attitudes & Values | Overall experience with AI |
|  |  |  | Evaluation of AI Use | Satisfaction with time needed |
|  |  |  | Reliability | Accuracy of AI |
|  |  |  | Usability | Satisfaction with ease of use |
| Wong et al. | 29 radiation therapists / dosimetrists | 5-point survey | Evaluation of AI Use | Satifsaction with AI results |
|  |  |  | Reliability | Degree of edits performed on AI results |
| Wong et al. [9] | 7 radiologists | 5-point survey | Evaluation of AI Use | Impact of AI on diagnostic confidence  Impact of AI on efficiency  Satisfaction with AI  Usefulness of AI |
|  |  |  | Individual Work Organization | Use of AI |
|  |  |  | Usability | Usability |
| Zia et al. [10] | 26 radiologists | Survey | Individual Work Organization | Use of AI |

**References**

1. Carlile M, Hurt B, Hsiao A, Hogarth M, Longhurst CA, Dameff C. Deployment of Artificial Intelligence for Radiographic Diagnosis of Covid‐19 Pneumonia in the Emergency Department. J Am Coll Emerg Physicians Open 2020;1(6):1459–1464. doi: 10.1002/emp2.12297

2. Cheikh AB, Gorincour G, Nivet H, May J, Seux M, Calame P, Thomson V, Delabrousse E, Crombé A. How Artificial Intelligence Improves Radiological Interpretation in Suspected Pulmonary Embolism. Eur Radiol 2022;32(9):5831–5842. doi: 10.1007/s00330-022-08645-2

3. Diao K, Chen Y, Liu Y, Chen B, Li W, Zhang L, YL Q, Zhang T, Zhang Y, Wu M, Li K, Song B. Diagnostic Study on Clinical Feasibility of an AI-Based Diagnostic System as a Second Reader on Mobile CT Images: A Preliminary Result. Ann Transl Med 2022;10(12):668. doi: 10.21037/atm-22-2157

4. Jones OT, Calanzani N, Saji S, Duffy SW, Emery J, Hamilton W, Singh H, Wit NJ de, Walter FM. Artificial Intelligence Techniques That May Be Applied to Primary Care Data to Facilitate Earlier Diagnosis of Cancer: Systematic Review. J Med Internet Res 2021;23(3):e23483. doi: 10.2196/23483

5. Marwaha A, Chitayat D, Meyn M, Mendoza-Londono R, Chad L. The Point-of-Care Use of a Facial Phenotyping Tool in the Genetics Clinic: Enhancing Diagnosis and Education with Machine Learning. Am J Med Genet A 2021 Apr;185(4):1151–1158. doi: 10.1002/ajmg.a.62092

6. Mueller FC, Raaschou H, Akhtar N, Brejnebol M, Collatz L, Andersen MB. Impact of Concurrent Use of Artificial Intelligence Tools on Radiologists Reading Time: A Prospective Feasibility Study. Acad Radiol 2022;29(7):1085–1090.

7. Nehme F, Coronel E, Barringer DA, Romero LG, Shafi MA, Ross WA, Ge PS. Performance and attitudes toward real-time computer-aided polyp detection during colonoscopy in a large tertiary referral center in the United States. Gastrointest Endosc 2023;98(1):100-109.e6. doi: https://doi.org/10.1016/j.gie.2023.02.016

8. Ruamviboonsuk P, Tiwari R, Sayres R, Nganthavee V, Hemarat K, Kongprayoon A, Raman R, Levinstein B, Liu Y, Schaekermann M, Lee R, Virmani S, Widner K, Chambers J, Hersch F, Peng L, Webster DR. Real-Time Diabetic Retinopathy Screening by Deep Learning in a Multisite National Screening Programme: A Prospective Interventional Cohort Study. Lancet Digit Health 2022 Apr;4(4). doi: 10.1016/S2589-7500(22)00017-6

9. Wong K, Homer S, Wei S, Yaghmai N, Estrada Paz O, Young T, Buhr R, Barjaktarevic I, Shrestha L, Daly M, Goldin J, Enzmann D, Brown M. Integration and evaluation of chest X-ray artificial intelligence in clinical practice. J Med Imaging Bellingham Wash 2023 Sep;10(5):051805. doi: 10.1117/1.JMI.10.5.051805

10. Zia A, Fletcher C, Bigwood S, Ratnakanthan P, Seah J, Lee R, Kavnoudias H, Law M. Retrospective analysis and prospective validation of an AI-based software for intracranial haemorrhage detection at a high-volume trauma centre. Sci Rep 2022 Nov;12(1):19885. doi: 10.1038/s41598-022-24504-y
