# Supplementary material for: Facilitators and Barriers to Implementing AI in Routine Medical Imaging: Systematic Review and Qualitative Analysis
Source: J Med Internet Res. 2025 Jul 21;27:e63649. doi: 10.2196/63649 (PMC12322614; doi:10.2196/63649)
Supplement: Multimedia Appendix 6 [file jmir_v27i1e63649_app6.docx]

### **Multimedia Appendix 6. Details on the extracted themes.**

Based on qualitative analyses of the studies included, we identified 180 statements from the publications that describe factors affecting the implementation of AI in clinical practice. These statements were organized into 12 main categories, and within each dimension, we grouped the recurring themes accordingly. Table 5 presents the example quotations per theme and which study mentioned the theme as a facilitator and/or barrier.

For the reference list, please consult this list of studies:

Arbabshirani et al. [1]; Batra et al. [2]; Carlile et al. [3]; Cha et al. [4]; Cheikh et al. [5]; Chen et al. [6]; Conant et al. [7]; Davis et al. [8]; Diao et al. [9]; Duron et al. [10]; Elijovich et al. [11]; Ginat [12]; Hassan et al. [13]; Jones et al. [14]; Ladabaum et al. [15]; Levy et al. [16]; Marwaha et al. [17]; Mueller et al. [18]; Nehme et al. [19]; Oppenheimer et al. [20]; Pierce et al. [21]; Potrezke et al. [22]; Quan et al. [23]; Raya-Povedano et al. [24]; Ruamviboonsuk et al. [25]; Sandbank et al. [26]; Schmuelling et al. [27]; Seyam et al. [28]; Tchou et al. [29]; Tricarico et al. [30]; Vassallo et al. [31]; Wang et al. [32]; Wang et al. [33]; Wittenberg et al. [34]; Wong et al. [35]; Wong et al. [36]; Yang et al. [37]; Zia et al. [38]

Table 6 Details on extracted themes

| **Dimension** | **Theme** | **Example** | **Number of Codes** | | **Classification** | **LoI** | **Study** | **Codes in Study** |
| --- | --- | --- | --- | --- | --- | --- | --- | --- |
|  |  |  | **B** | **F** |  |  |  |  |
| Evaluation of AI Use | Usefulness | “There is a consensus among radiologists and trainees that the tool is both accurate and helpful during the regular workday […]” (Pierce et al. [21] p. 1504) | 12 | 16 | B | External | Vassallo et al. [31] | 1 |
|  |  |  |  |  | B | External | Tchou et al. [29] | 1 |
|  |  |  |  |  | B | Initial | Jones et al. [14] | 2 |
|  |  |  |  |  | B | Initial | Nehme et al. [19] | 3 |
|  |  |  |  |  | B | Initial | Wong et al. [36] | 1 |
|  |  |  |  |  | B | Full | Ruamviboonsuk et al. [25] | 1 |
|  |  |  |  |  | B | Full | Pierce et al. [21] | 1 |
|  |  |  |  |  | B | Full | Mueller et al. [18] | 2 |
|  |  |  |  |  | F | External | Conant et al. [7] | 1 |
|  |  |  |  |  | F | Initial | Cheikh et al. [5] | 1 |
|  |  |  |  |  | F | Initial | Marwaha et al. [17] | 1 |
|  |  |  |  |  | F | Initial | Potrezke et al. [22] | 1 |
|  |  |  |  |  | F | Initial | Wong et al. [36] | 1 |
|  |  |  |  |  | F | Initial | Jones et al. [14] | 2 |
|  |  |  |  |  | F | Full | Cha et al. [4] | 2 |
|  |  |  |  |  | F | Full | Hassan et al. [13] | 1 |
|  |  |  |  |  | F | Full | Mueller et al. [18] | 1 |
|  |  |  |  |  | F | Full | Oppenheimer et al. [20] | 1 |
|  |  |  |  |  | F | Full | Pierce et al. [21] | 1 |
|  |  |  |  |  | F | Full | Ruamviboonsuk et al. [25] | 3 |
|  | Impact on Decision-making | “Indeed, radiologists stressed the importance of AI to strengthen their conclusions, especially to confirm negative findings […]” (Cheikh et al. [5] p. 5840) | 5 | 4 | B | Initial | Zia et al. [38] | 1 |
|  |  |  |  |  | B | Initial | Ladabaum et al. [15] | 1 |
|  |  |  |  |  | B | Full | Levy et al. [16] | 1 |
|  |  |  |  |  | B | Full | Oppenheimer et al. [20] | 1 |
|  |  |  |  |  | B | Full | Seyam et al. [28] | 1 |
|  |  |  |  |  | F | Initial | Cheikh et al. [5] | 1 |
|  |  |  |  |  | F | Initial | Nehme et al. [19] | 1 |
|  |  |  |  |  | F | Initial | Potrezke et al. [22] | 1 |
|  |  |  |  |  | F | Full | Sandbank et al. [26] | 1 |
| Fit into the Workflow | Accessibility of Results | “As the AI results are directly shown in our clinical PACS system, the board-certified radiologist was able to see the AI results while correcting the initial report […]” (Oppenheimer et al. [20] p. 8) | 2 | 10 | B | Initial | Nehme et al. [19] | 1 |
|  |  |  |  |  | B | Initial | Zia et al. [38] | 1 |
|  |  |  |  |  | F | Initial | Ginat [12] | 1 |
|  |  |  |  |  | F | Initial | Carlile et al. [3] | 1 |
|  |  |  |  |  | F | Initial | Tricarico et al. [30] | 1 |
|  |  |  |  |  | F | Full | Davis et al. [8] | 2 |
|  |  |  |  |  | F | Full | Elijovich et al. [11] | 1 |
|  |  |  |  |  | F | Full | Hassan et al. [13] | 1 |
|  |  |  |  |  | F | Full | Oppenheimer et al. [20] | 1 |
|  |  |  |  |  | F | Full | Pierce et al. [21] | 1 |
|  |  |  |  |  | F | Full | Mueller et al. [18] | 1 |
|  | Data Processing | “All urgent, non-contrast head CT scans […] were automatically and immediately forwarded for analysis by the software in real-time.” (Ginat [12] p. 2) | 0 | 5 | F | External | Vassallo et al. [31] | 1 |
|  |  |  |  |  | F | Initial | Ginat [12] | 1 |
|  |  |  |  |  | F | Full | Arbabshirani et al. [1] | 1 |
|  |  |  |  |  | F | Full | Batra et al. [2] | 1 |
|  |  |  |  |  | F | Full | Elijovich et al. [11] | 1 |
|  | Distraction | “Many physicians believed it had too many false-positive signals, was too distracting […], and had an audio beep that was too loud when a polyp was discovered.” (Nehme et al. [19] p. 107) | 5 | 0 | B | External | Wang et al. [32] | 1 |
|  |  |  |  |  | B | Initial | Nehme et al. [19] | 3 |
|  |  |  |  |  | B | Full | Quan et al. [23] | 1 |
|  | No Disruptions due to AI | “Furthermore, the detection delay was hardly noticeable for endoscopists.” (Wang et al. [32] p. 1814) | 0 | 5 | F | External | Wang et al. [32] | 1 |
|  |  |  |  |  | F | Full | Davis et al. [8] | 1 |
|  |  |  |  |  | F | Full | Pierce et al. [21] | 1 |
|  |  |  |  |  | F | Full | Seyam et al. [28] | 1 |
|  |  |  |  |  | F | Full | Quan et al. [23] | 1 |
|  | Additional Worksteps | “The AI tool in the current study generates an additional image series for review and thus could conceivably decrease or increase interpretation time.” (Batra et al. [2] p. 8) | 1 | 1 | B | External | Batra et al. [2] | 1 |
|  |  |  |  |  | F | Initial | Batra et al. [2] | 1 |
| Imple-mentation Procedure | Deployment Strategy | “However, a minimalist deployment strategy may not ensure success.“ (Ladabaum et al. [15] p. 483) | 3 | 5 | B | Initial | Potrezke et al. [22] | 1 |
|  |  |  |  |  | B | Initial | Ladabaum et al. [15] | 2 |
|  |  |  |  |  | F | Initial | Ladabaum et al. [15] | 1 |
|  |  |  |  |  | F | Initial | Potrezke et al. [22] | 1 |
|  |  |  |  |  | F | Full | Pierce et al. [21] | 3 |
|  | Technology Readiness | “To increase user awareness and buy-in of these new features, we began a campaign to educate users throughout the radiology department before full deployment.“ (Pierce et al. [21] p. 1500) | 3 | 2 | B | Initial | Marwaha et al. [17] | 1 |
|  |  |  |  |  | B | Full | Ruamviboonsuk et al. [25] | 2 |
|  |  |  |  |  | F | Initial | Potrezke et al. [22] | 1 |
|  |  |  |  |  | F | Full | Pierce et al. [21] | 1 |
|  | Training of Users | “There were 5 participating radiologists who were trained and instructed on the implementation of the software.“ (Ginat [12] p. 2) | 0 | 5 | F | External | Chen et al. [6] | 1 |
|  |  |  |  |  | F | External | Conant et al. [7] | 1 |
|  |  |  |  |  | F | Initial | Ginat [12] | 1 |
|  |  |  |  |  | F | Initial | Jones et al. [14] | 1 |
|  |  |  |  |  | F | Initial | Ladabaum et al. [15] | 1 |
|  | Continuous Maintenance | “The team met weekly post-deployment to monitor the effectiveness of the system and provide input on the correctness, usefulness, presentation, and frequency of alerts.” (Wong et al. [36] p. 10) | 0 | 3 | F | Initial | Wong et al. [36] | 2 |
|  |  |  |  |  | F | Initial | Zia et al. [38] | 1 |
|  | Internal Testing | “After the upgrade, no study readings were performed for  2 weeks so that the radiologists could become familiar with the new system and to allow time for any initial issues to be corrected.“ (Tchou et al. [29] p. 42) | 1 | 2 | B | Full | Ruamviboonsuk et al. [25] | 1 |
|  |  |  |  |  | F | External | Tchou et al. [29] | 1 |
|  |  |  |  |  | F | Full | Pierce et al. [21] | 1 |
| Explainability of AI | Visualization | “In the case of positive AI results, a color-encoded map was transferred into Imadis PACS (re-identification process) to enable visualization of the area suspected.“ (Cheikh et al. [5] p. 5833) | 1 | 6 | B | Full | Arbabshirani et al. [1] | 1 |
|  |  |  |  |  | F | External | Conant et al. [7] | 1 |
|  |  |  |  |  | F | External | Duron et al. [10] | 1 |
|  |  |  |  |  | F | Initial | Cheikh et al. [5] | 1 |
|  |  |  |  |  | F | Initial | Tricarico et al. [30] | 1 |
|  |  |  |  |  | F | Initial | Jones et al. [14] | 1 |
|  |  |  |  |  | F | Full | Mueller et al. [18] | 1 |
|  | Confidence Score | “For each case, the model provided a list of suggested findings, listed as ‘priority’ or ‘other’, along with a confidence indicator.“ (Jones et al. [14] p. 3) | 0 | 3 | F | External | Conant et al. [7] | 1 |
|  |  |  |  |  | F | External | Duron et al. [10] | 1 |
|  |  |  |  |  | F | Initial | Jones et al. [14] | 1 |
|  | Trans-parency | “The disagreement between radiologists and the AI algorithm regarding HR [high risk] nodule diagnosis implied that the AI algorithm “thinks” in a different way from human beings.“ (Diao et al. [9] p. 9) | 2 | 1 | B | Initial | Diao et al. [9] | 1 |
|  |  |  |  |  | B | Full | Arbabshirani et al. [1] | 1 |
|  |  |  |  |  | F | Initial | Diao et al. [9] | 1 |
| Attitudes & Values | Attitude Towards AI | “Nine radiologists (90%) demonstrated an improved attitude towards the use of the AI diagnostic viewer by the end of the study.“ (Jones et al. [14] p. 7) | 0 | 7 | F | Initial | Jones et al. [14] | 3 |
|  |  |  |  |  | F | Initial | Ladabaum et al. [15] | 1 |
|  |  |  |  |  | F | Initial | Marwaha et al. [17] | 1 |
|  |  |  |  |  | F | Initial | Nehme et al. [19] | 2 |
|  | Expectations of AI Use | “[…] 81.3% of physicians and 62.5% of staff expected a longer procedure time with Al-assisted colonoscopy.” (Nehme et al. [19] p. 102) | 2 | 3 | B | Initial | Nehme et al. [19] | 2 |
|  |  |  |  |  | F | Initial | Nehme et al. [19] | 2 |
|  |  |  |  |  | F | Initial | Potrezke et al. [22] | 1 |
| Inter-operability | Integration with Local System | “Features such as worklist prioritization and context matching were requested by the radiologists to improve their workflow but were not achievable with the institution’s PACS/RIS.“ (Zia et al. [38] p. 6) | 4 | 6 | B | External | Raya-Povedano et al. [24] | 1 |
|  |  |  |  |  | B | Initial | Zia et al. [38] | 2 |
|  |  |  |  |  | B | Full | Ruamviboonsuk et al. [25] | 1 |
|  |  |  |  |  | F | Initial | Tricarico et al. [30] | 1 |
|  |  |  |  |  | F | Full | Mueller et al. [18] | 1 |
|  |  |  |  |  | F | Full | Oppenheimer et al. [20] | 1 |
|  |  |  |  |  | F | Full | Pierce et al. [21] | 3 |
|  | Flexibility on Input Data | “However, the IdxDR [AI tool] can now use images from other types of fundus cameras in Europe and other regions worldwide.” (Yang et al. [37] p. 7) | 0 | 2 | F | Initial | Yang et al. [37] | 1 |
|  |  |  |  |  | F | Full | Ruamviboonsuk et al. [25] | 1 |
| Stakeholder Involvement | User Engagement in Implementation | “Educational materials were developed for each role. Learning modules were available electronically and included both text and graphic presentation of the background, rationale, and steps involved for algorithm implementation.“ (Potretzke et al. [22] p. 697) | 2 | 3 | B | Initial | Ladabaum et al. [15] | 1 |
|  |  |  |  |  | B | Initial | Zia et al. [38] | 1 |
|  |  |  |  |  | F | Initial | Potrezke et al. [22] | 3 |
|  | Stakeholder Discussion | “This was a department-wide effort with multiple discussions between administrators, technologists, managers, residents, and attending radiologists.“ (Pierce et al. [21] p. 1500) | 0 | 3 | F | Full | Elijovich et al. [11] | 1 |
|  |  |  |  |  | F | Full | Pierce et al. [21] | 2 |
|  | Stakeholder Testing | “The expert team should check for clinical relevance, usefulness of all the alerts and ensure that they can be acted on. […]“ (Wong et al. [36] p. 10) | 0 | 2 | F | Initial | Wong et al. [36] | 1 |
|  |  |  |  |  | F | Full | Pierce et al. [21] | 1 |
|  | Collaboration with Vendors | “Additionally, in hindsight, the compatibility of our practice’s underlying PACS infrastructure and willingness of partnered vendors to collaborate with us were both crucial factors during the development of this workflow for pneumothorax detection.“ (Pierce et al. [21] p. 1503) | 0 | 1 | F | Full | Pierce et al. [21] | 1 |
|  | User Engagement in Design | “This visualization has been defined together with the radiologists to minimize the cognitive overhead introduced by the introduction of the AIppo [AI tool] prediction score in the workflow.“ (Tricarico et al. [30] p. 7) | 0 | 1 | F | Initial | Tricarico et al. [30] | 1 |
| Usability | Ease of Use | “Overall, 86% either strongly agreed or somewhat agreed that the tool was easy to use in the existing workflow.“ (Carlile et al. [3] p. 1461) | 2 | 3 | B | External | Conant et al. [7] | 1 |
|  |  |  |  |  | B | Initial | Marwaha et al. [17] | 1 |
|  |  |  |  |  | F | Initial | Carlile et al. [3] | 1 |
|  |  |  |  |  | F | Initial | Jones et al. [14] | 1 |
|  |  |  |  |  | F | Full | Quan et al. [23] | 1 |
|  | Standalone Use | “No automated contour sets were deemed acceptable without edits […].” (Cha et al. [4] p. 6) | 2 | 1 | B | Full | Elijovich et al. [11] | 1 |
|  |  |  |  |  | B | Full | Cha et al. [4] | 1 |
|  |  |  |  |  | F | Full | Wong et al. [35] | 1 |
|  | Educational Value | “Trainees appreciated the use in the software as an educational tool […]” (Marwaha et al. [17] p. 1154) | 0 | 2 | F | Initial | Marwaha et al. [17] | 2 |
|  | Future Potential | “A high proportion of responding physicians (64.7%) and staff (76.9%) believed there is a strong role for AI in colonoscopy […].” (Nehme et al. [19] p. 104) | 0 | 2 | F | Initial | Nehme et al. [19] | 2 |
| Reliability | Sensitivity | “The AI software offered a handful of instances with results that could best be described as “obvious mistakes”. (Oppenheimer et al. [20] p. 8) | 6 | 1 | B | External | Wang et al. [32] | 1 |
|  |  |  |  |  | B | External | Wittenberg et al. [34] | 1 |
|  |  |  |  |  | B | Initial | Jones et al. [14] | 1 |
|  |  |  |  |  | B | Full | Mueller et al. [18] | 1 |
|  |  |  |  |  | B | Full | Oppenheimer et al. [20] | 1 |
|  |  |  |  |  | B | Full | Elijovich et al. [11] | 1 |
|  |  |  |  |  | F | Full | Hassan et al. [13] | 1 |
|  | Automatic Transfer | “It is important to recognize that the AI algorithm never failed to transmit an alert.” (Elijovic et al. [11] p. 707) | 1 | 1 | B |  | Batra et al. [2] | 1 |
|  |  |  |  |  | F | Full | Elijovich et al. [11] | 1 |
|  | Development | “First, the algorithm was trained on data from Tongji Hospital only, which could compromise the robustness of the algorithm […].” (Wang et al. [33] p. e514) | 2 | 0 | B | External | Wang et al. [33] | 1 |
|  |  |  |  |  | B | Full | Schmuelling et al. [27] | 1 |
| Individual Work organization | Choice of Interaction | “The ML tool used here encourages this process by seamlessly integrating into the workflow and allowing the radiologist to drive the level of interaction.“ (Davis et al. [8] p. 560) | 2 | 3 | B | Initial | Nehme et al. [19] | 1 |
|  |  |  |  |  | B | Initial | Zia et al. [38] | 1 |
|  |  |  |  |  | F | Full | Davis et al. [8] | 3 |
|  | Specific Work Order Preference | “Even after discovering that the ML system had flagged a false positive, efficiency dictates that our radiologists would have been more likely to dictate the case rather than close the examination and move on to a different one.“ (Davis et al. [8] p. 560) | 1 | 1 | B | Full | Davis et al. [8] | 1 |
|  |  |  |  |  | F | Initial | Ginat [12] | 1 |
| Impact on the Role of Clinicians | Replacement | “In short, the algorithm did not replace physician decision-making but, rather, was intended to be another data point […] to consider in context.” (Carlile et al. [3] p. 463) | 1 | 3 | B | Initial | Jones et al. [14] | 1 |
|  |  |  |  |  | F | Initial | Carlile et al. [3] | 1 |
|  |  |  |  |  | F | Initial | Jones et al. [14] | 1 |
|  |  |  |  |  | F | Full | Elijovich et al. [11] | 1 |
|  | No Automation Bias | “Interestingly, we did not see a reduction in the magnitude of physician contour edits over time, […] a phenomenon called automation bias […].” (Cha et al. [4] p. 7) | 0 | 2 | F | Full | Cha et al. [4] | 1 |
|  |  |  |  |  | F | Full | Pierce et al. [21] | 1 |
| Medicolegal Concerns | Data Privacy Concerns | “Finally, there were staff concerns noted over perceived accuracy, ease of use, the consenting process, and the privacy of patient data.” (Marwaha et al. [17] p. 1154) | 3 | 0 | B | Initial | Marwaha et al. [17] | 3 |
|  | Legal Implications | “Finally, although our results suggest that no human reading of low suspicion examinations would be the most optimal […] further legal discussions would be needed to establish a framework where this strategy is safe for all the parties involved in screening.” (Raya-Povedano et al. [24] p. 64) | 2 | 0 | B | External | Raya-Povedano et al. [24] | 1 |
|  |  |  |  |  | B | Full | Seyam et al. [28] | 1 |

**Notes:** Abbreviations: F Facilitator(s); B Barrier(s); LoI Level of Implementation

**References**

1. Arbabshirani MR, Fornwalt BK, Mongelluzzo GJ, Suever JD, Geise BD, Patel AA, Brandon K.; ORCID: https://orcid.org/0000-0002-6231-9442 MGJA-F. Advanced Machine Learning in Action: Identification of Intracranial Hemorrhage on Computed Tomography Scans of the Head with Clinical Workflow Integration. Npj Digit Med 2018;1(1):9. doi: 10.1038/s41746-017-0015-z

2. Batra K, Xi Y, Bhagwat S, Espino A, Peshock R. Radiologist Worklist Reprioritization Using Artificial Intelligence: Impact on Report Turnaround Times for CTPA Examinations Positive for Acute Pulmonary Embolism. AJR Am J Roentgenol 2023 Apr; doi: 10.2214/AJR.22.28949

3. Carlile M, Hurt B, Hsiao A, Hogarth M, Longhurst CA, Dameff C. Deployment of Artificial Intelligence for Radiographic Diagnosis of Covid‐19 Pneumonia in the Emergency Department. J Am Coll Emerg Physicians Open 2020 Dec;1(6):1459–1464. doi: 10.1002/emp2.12297

4. Cha E, Elguindi S, Onochie I, Gorovets D, Deasy JO, Zelefsky M, Gillespie EF. Clinical Implementation of Deep Learning Contour Autosegmentation for Prostate Radiotherapy. Radiother Oncol 2021;159:1–7. doi: 10.1016/j.radonc.2021.02.040

5. Cheikh AB, Gorincour G, Nivet H, May J, Seux M, Calame P, Thomson V, Delabrousse E, Crombé A. How Artificial Intelligence Improves Radiological Interpretation in Suspected Pulmonary Embolism. Eur Radiol 2022 Mar 22;32(9):5831–5842. doi: 10.1007/s00330-022-08645-2

6. Chen W, Wu J, Wei R, Wu S, Xia C, Wang D, Liu D, Zheng L, Zou T, Li R, Qi X, Zhang X. Improving the Diagnosis of Acute Ischemic Stroke on Non-Contrast Ct Using Deep Learning: A Multicenter Study. Insights Imaging 2022 Dec;13(1):184. doi: 10.1186/s13244-022-01331-3

7. Conant EF, Toledano AY, Periaswamy S, Fotin SV, Go J, Boatsman JE, Hoffmeister JW. Improving Accuracy and Efficiency with Concurrent Use of Artificial Intelligence for Digital Breast Tomosynthesis. Radiol Artif Intell 2019 Jul;1(4):e180096. doi: 10.1148/ryai.2019180096

8. Davis MA, Rao B, Cedeno PA, Saha A, Zohrabian VM. Machine Learning and Improved Quality Metrics in Acute Intracranial Hemorrhage by Noncontrast Computed Tomography. Curr Probl Diagn Radiol 2022 Jul;51(4):556–561. doi: 10.1067/j.cpradiol.2020.10.007

9. Diao K, Chen Y, Liu Y, Chen B, Li W, Zhang L, YL Q, Zhang T, Zhang Y, Wu M, Li K, Song B. Diagnostic Study on Clinical Feasibility of an AI-Based Diagnostic System as a Second Reader on Mobile CT Images: A Preliminary Result. Ann Transl Med 2022 Jun;10(12):668. doi: 10.21037/atm-22-2157

10. Duron L, Ducarouge A, Gillibert A, Lainé J, Allouche C, Cherel N, Zhang Z, Nitche N, Lacave E, Pourchot A, Felter A, Lassalle L, Regnard N-E, Feydy A. Assessment of an AI Aid in Detection of Adult Appendicular Skeletal Fractures by Emergency Physicians and Radiologists: A Multicenter Cross-sectional Diagnostic Study. Radiology 2021 Jul;300(1):120–129. doi: 10.1148/radiol.2021203886

11. Elijovich L, Dornbos III D, Nickele C, Alexandrov A, Inoa-Acosta V, Arthur AS, Hoit D. Automated Emergent Large Vessel Occlusion Detection by Artificial Intelligence Improves Stroke Workflow in a Hub and Spoke Stroke System of Care. J NeuroInterventional Surg 2022 Jul;14(7):704–708. doi: 10.1136/neurintsurg-2021-017714

12. Ginat D. Implementation of Machine Learning Software on the Radiology Worklist Decreases Scan View Delay for the Detection of Intracranial Hemorrhage on CT. Brain Sci D. Ginat, Department of Radiology, University of Chicago, Chicago, IL 60615, United States. E-mail: dginat@radiology.bsd.uchicago.edu Switzerland: MDPI; 2021;11(7):832. doi: 10.3390/brainsci11070832

13. Hassan A, Ringheanu V, Tekle W. The Implementation of Artificial Intelligence Significantly Reduces Door-in-Door-Out Times in a Primary Care Center Prior to Transfer. Interv Neuroradiol J Peritherapeutic Neuroradiol Surg Proced Relat Neurosci 2022 Aug;15910199221122848. doi: 10.1177/15910199221122848

14. Jones CM, Danaher L, Milne MR, Tang C, Seah J, Oakden-Rayner L, Johnson A, Buchlak QD, Esmaili N. Assessment of the Effect of a Comprehensive Chest Radiograph Deep Learning Model on Radiologist Reports and Patient Outcomes: A Real-World Observational Study. BMJ Open 2021 Dec;11(12):e052902. doi: 10.1136/bmjopen-2021-052902

15. Ladabaum U, Shepard J, Weng Y, Desai M, Singer SJ, Mannalithara A. Computer-Aided Detection of Polyps Does Not Improve Colonoscopist Performance in a Pragmatic Implementation Trial. Gastroenterology 2023 Mar;164(3). doi: 10.1053/j.gastro.2022.12.004

16. Levy I, Bruckmayer L, Klang E, Ben-Horin S, Kopylov U. Artificial Intelligence-Aided Colonoscopy Does Not Increase Adenoma Detection Rate in Routine Clinical Practice. Am J Gastroenterol 2022 Nov;117(11):1871–1873. doi: 10.14309/ajg.0000000000001970

17. Marwaha A, Chitayat D, Meyn M, Mendoza-Londono R, Chad L. The Point-of-Care Use of a Facial Phenotyping Tool in the Genetics Clinic: Enhancing Diagnosis and Education with Machine Learning. Am J Med Genet A 2021 Apr;185(4):1151–1158. doi: 10.1002/ajmg.a.62092

18. Mueller FC, Raaschou H, Akhtar N, Brejnebol M, Collatz L, Andersen MB. Impact of Concurrent Use of Artificial Intelligence Tools on Radiologists Reading Time: A Prospective Feasibility Study. Acad Radiol 2022;29(7):1085–1090. doi: 10.1016/j.acra.2021.10.008

19. Nehme F, Coronel E, Barringer D, Romero L, Shafi M, Ross W, Ge P. Performance and Attitudes Toward Real-time Computer-aided Polyp Detection during Colonoscopy in a Large Tertiary Referral Center in the United States. Gastrointest Endosc 2023 Feb; doi: 10.1016/j.gie.2023.02.016

20. Oppenheimer J, Lüken S, Hamm B, Niehues S. A Prospective Approach to Integration of AI Fracture Detection Software in Radiographs into Clinical Workflow. Life Basel Switz 2023 Jan;13(1). doi: 10.3390/life13010223

21. Pierce J, Rosipko B, Youngblood L, Gilkeson R, Gupta A, Bittencourt L. Seamless Integration of Artificial Intelligence Into the Clinical Environment: Our Experience With a Novel Pneumothorax Detection Artificial Intelligence Algorithm. J Am Coll Radiol JACR 2021 Nov;18(11):1497–1505.

22. Potretzke T, Korfiatis P, Blezek D, Edwards M, Klug J, Cook C, Gregory A, Harris P, Chebib F, Hogan M, Torres V, Bolan C, Sandrasegaran K, Kawashima A, Collins J, Takahashi N, Hartman R, Williamson E, King B, Callstrom M, Erickson B, Kline T. Clinical Implementation of an Artificial Intelligence Algorithm for Magnetic Resonance-Derived Measurement of Total Kidney Volume. Mayo Clin Proc 2023 May;98(5):689–700. doi: 10.1016/j.mayocp.2022.12.019

23. Quan SY, Wei MT, Lee J, Mohi-Ud-Din R, Mostaghim R, Sachdev R, Siegel D, Friedlander Y, Friedland S. Clinical Evaluation of a Real-Time Artificial Intelligence-Based Polyp Detection System: A US Multi-Center Pilot Study. Sci Rep 2022 Apr 21;12(1):6598. doi: 10.1038/s41598-022-10597-y

24. Raya-Povedano JL, Romero-Martín S, Elías-Cabot E, Gubern-Mérida A, Rodríguez-Ruiz A, Álvarez-Benito M. AI-based Strategies to Reduce Workload in Breast Cancer Screening with Mammography and Tomosynthesis: A Retrospective Evaluation. Radiology 2021 Jul;300(1):57–65. doi: 10.1148/radiol.2021203555

25. Ruamviboonsuk P, Tiwari R, Sayres R, Nganthavee V, Hemarat K, Kongprayoon A, Raman R, Levinstein B, Liu Y, Schaekermann M, Lee R, Virmani S, Widner K, Chambers J, Hersch F, Peng L, Webster DR. Real-Time Diabetic Retinopathy Screening by Deep Learning in a Multisite National Screening Programme: A Prospective Interventional Cohort Study. Lancet Digit Health 2022 Apr;4(4). doi: 10.1016/S2589-7500(22)00017-6

26. Sandbank J, Bataillon G, Nudelman A, Krasnitsky I, Mikulinsky R, Bien L, Thibault L, Albrecht Shach A, Sebag G, Clark D, Laifenfeld D, Schnitt S, Linhart C, Vecsler M, Vincent-Salomon A. Validation and Real-World Clinical Application of an Artificial Intelligence Algorithm for Breast Cancer Detection in Biopsies. Npj Breast Cancer 2022 Dec;8(1):129. doi: 10.1038/s41523-022-00496-w

27. Schmuelling L, Franzeck FC, Nickel CH, Mansella G, Bingisser R, Schmidt N, Stieltjes B, Bremerich J, Sauter AW, Weikert T, Sommer G. Deep Learning-Based Automated Detection of Pulmonary Embolism on CT Pulmonary Angiograms: No Significant Effects on Report Communication Times and Patient Turnaround in the Emergency Department Nine Months After Technical Implementation. Eur J Radiol 2021 Aug;141:109816. doi: 10.1016/j.ejrad.2021.109816

28. Seyam M, Weikert T, Sauter A, Brehm A, Psychogios M-N, Blackham KA. Utilization of Artificial Intelligence-based Intracranial Hemorrhage Detection on Emergent Noncontrast CT Images in Clinical Workflow. Radiol Artif Intell 2022;4(2):e210168.

29. Tchou PM, Haygood TM, Atkinson EN, Stephens TW, Davis PL, Arribas EM, Geiser WR, Whitman GJ. Interpretation Time of Computer-aided Detection at Screening Mammography. Radiology 2010 Oct;257(1):40–46. doi: 10.1148/radiol.10092170

30. Tricarico D, Calandri M, Barba M, Piatti C, Geninatti C, Basile D, Gatti M, Melis M, Veltri A. Convolutional Neural Network-Based Automatic Analysis of Chest Radiographs for the Detection of COVID-19 Pneumonia: A Prioritizing Tool in the Emergency Department, Phase I Study and Preliminary “Real Life” Results. Diagnostics 2022;12(3):570. doi: https://dx.doi.org/10.3390/diagnostics12030570 PT - Article

31. Vassallo L, Traverso A, Agnello M, Bracco C, Campanella D, Chiara G, Fantacci ME, Lopez Torres E, Manca A, Saletta M, Giannini V, Mazzetti S, Stasi M, Cerello P, Regge D. A Cloud-Based Computer-Aided Detection System Improves Identification of Lung Nodules on Computed Tomography Scans of Patients with Extra-Thoracic Malignancies. Eur Radiol 2019 Jan;29(1):144–152. doi: 10.1007/s00330-018-5528-6

32. Wang P, Berzin TM, Glissen Brown JR, Bharadwaj S, Becq A, Xiao X, Liu P, Li L, Song Y, Zhang D, Li Y, Xu G, Tu M, Liu X. Real-Time Automatic Detection System Increases Colonoscopic Polyp and Adenoma Detection Rates: A Prospective Randomised Controlled Study. Gut 2019 Oct;68(10):1813–1819. doi: 10.1136/gutjnl-2018-317500

33. Wang M, Xia C, Huang L, Xu S, Qin C, Liu J, Cao Y, Yu P, Zhu T, Zhu H, Wu C, Zhang R, Chen X, Wang J, Du G, Zhang C, Wang S, Chen K, Liu Z, Xia L, Wang W. Deep Learning-Based Triage and Analysis of Lesion Burden for Covid-19: A Retrospective Study with External Validation. Lancet Digit Health 2020 Oct;2(10):e506–e515. doi: 10.1016/S2589-7500(20)30199-0

34. Wittenberg R, Berger FH, Peters JF, Weber M, van Hoorn F, Beenen LFM, van Doorn MMAC, van Schuppen J, Zijlstra IjAJ, Prokop M, Schaefer-Prokop CM. Acute Pulmonary Embolism: Effect of a Computer-assisted Detection Prototype on Diagnosis—An Observer Study. Radiology 2012 Jan;262(1):305–313. doi: 10.1148/radiol.11110372

35. Wong J, Huang V, Wells D, Giambattista J, Giambattista J, Kolbeck C, Otto K, Saibishkumar EP, Alexander A. Implementation of Deep Learning-Based Auto-Segmentation for Radiotherapy Planning Structures: A Workflow Study at Two Cancer Centers. Radiat Oncol 2021 Dec;16(1):101. doi: 10.1186/s13014-021-01831-4

36. Wong K, Homer S, Wei S, Yaghmai N, Estrada Paz O, Young T, Buhr R, Barjaktarevic I, Shrestha L, Daly M, Goldin J, Enzmann D, Brown M. Integration and Evaluation of Chest X-Ray Artificial Intelligence in Clinical Practice. J Med Imaging 2023 Sep;10(5):051805. doi: 10.1117/1.JMI.10.5.051805

37. Yang Y, Pan J, Yuan M, Lai K, Xie H, Ma L, Xu S, Deng R, Zhao M, Luo Y, Lin X. Performance of the AIDRScreening System in Detecting Diabetic Retinopathy in the Fundus Photographs of Chinese Patients: A Prospective, Multicenter, Clinical Study. Ann Transl Med 2022 Oct;10(20):1088. doi: 10.21037/atm-22-350

38. Zia A, Fletcher C, Bigwood S, Ratnakanthan P, Seah J, Lee R, Kavnoudias H, Law M. Retrospective Analysis and Prospective Validation of an Ai-Based Software for Intracranial Haemorrhage Detection at a High-Volume Trauma Centre. Sci Rep 2022 Nov;12(1):19885. doi: 10.1038/s41598-022-24504-y
