# Supplementary material for: Facilitators and Barriers to Implementing AI in Routine Medical Imaging: Systematic Review and Qualitative Analysis
Source: J Med Internet Res. 2025 Jul 21;27:e63649. doi: 10.2196/63649 (PMC12322614; doi:10.2196/63649)
Supplement: Multimedia Appendix 8 [file jmir_v27i1e63649_app8.docx]

**Multimedia Appendix 8. Moderators extracted from the included publications.**

We defined moderators as external factors which are independent of the AI too, but influenced its uptake or use. Table 7 presents the moderators with an example and the studies which named it.

For the reference list, please consult this list of studies:

Arbabshirani et al. [1]; Batra et al. [2]; Carlile et al. [3]; Cha et al. [4]; Cheikh et al. [5]; Chen et al. [6]; Conant et al. [7]; Davis et al. [8]; Diao et al. [9]; Duron et al. [10]; Elijovich et al. [11]; Ginat [12]; Hassan et al. [13]; Jones et al. [14]; Ladabaum et al. [15]; Levy et al. [16]; Marwaha et al. [17]; Mueller et al. [18]; Nehme et al. [19]; Oppenheimer et al. [20]; Pierce et al. [21]; Potrezke et al. [22]; Quan et al. [23]; Raya-Povedano et al. [24]; Ruamviboonsuk et al. [25]; Sandbank et al. [26]; Schmuelling et al. [27]; Seyam et al. [28]; Tchou et al. [29]; Tricarico et al. [30]; Vassallo et al. [31]; Wang et al. [32]; Wang et al. [33]; Wittenberg et al. [34]; Wong et al. [35]; Wong et al. [36]; Yang et al. [37]; Zia et al. [38]

Table 8 Moderating factors to AI use

| **Moderator** | **Count** | **Study** | **Level of Implementation** | **Example** |
| --- | --- | --- | --- | --- |
| Task | 10 | Tchou et al. [29] | External | “[…] the diagnosis provided by the AI algorithm was most useful when the target nodule had a larger volume, ground glass opacity (GGO), or recognized signs of malignancy.” (Diao et al. [9] p. 7) |
|  |  | Wang et al. [32] | External |  |
|  |  | Wittenberg et al. [34] | External |  |
|  |  | Diao et al. [9] | Initial |  |
|  |  | Ginat [12] | Initial |  |
|  |  | Jones et al. [14] | Initial |  |
|  |  | Nehme et al. [19] | Initial |  |
|  |  | Yang et al. [37] | Initial |  |
|  |  | Ruamviboonsuk et al. [25] | Full |  |
|  |  | Schmuelling et al. [27] | Full |  |
| Human Behavior | 8 | Wang et al. [32] | External | “A limitation of this study is that there was a small, but heterogeneous group of participating radiologists, […] each of whom could have different work habits.” (Ginat [12] p. 5) |
|  |  | Ginat [12] | Initial |  |
|  |  | Jones et al. [14] | Initial |  |
|  |  | Nehme et al. [19] | Initial |  |
|  |  | Cha et al. [4] | Full |  |
|  |  | Elijovich et al. [11] | Full |  |
|  |  | Levy et al. [16] | Full |  |
|  |  | Mueller et al. [18] | Full |  |
| Experience | 7 | Tchou et al. [29] | External | “In general, resident physicians responding to the survey reported they found the AI implementation easier to use than attendings […]” (Carlile et al. [3] p. 1461) |
|  |  | Carlile et al. [3] | Initial |  |
|  |  | Jones et al. [14] | Initial |  |
|  |  | Ladabaum et al. [15] | Initial |  |
|  |  | Nehme et al. [19] | Initial |  |
|  |  | Mueller et al. [18] | Full |  |
|  |  | Oppenheimer et al. [20] | Full |  |
| Setting | 4 | Ginat [12] | Initial | “Results during night and weekend shifts may change reader performance.” (Oppenheimer et al. [20] p. 8) |
|  |  | Wong et al. [36] | Initial |  |
|  |  | Oppenheimer et al. [20] | Full |  |
|  |  | Schmuelling et al. [27] | Full |  |

**References**

1. Arbabshirani MR, Fornwalt BK, Mongelluzzo GJ, Suever JD, Geise BD, Patel AA, Brandon K.; ORCID: https://orcid.org/0000-0002-6231-9442 MGJA-F. Advanced Machine Learning in Action: Identification of Intracranial Hemorrhage on Computed Tomography Scans of the Head with Clinical Workflow Integration. Npj Digit Med 2018;1(1):9. doi: 10.1038/s41746-017-0015-z

2. Batra K, Xi Y, Bhagwat S, Espino A, Peshock R. Radiologist Worklist Reprioritization Using Artificial Intelligence: Impact on Report Turnaround Times for CTPA Examinations Positive for Acute Pulmonary Embolism. AJR Am J Roentgenol 2023 Apr; doi: 10.2214/AJR.22.28949

3. Carlile M, Hurt B, Hsiao A, Hogarth M, Longhurst CA, Dameff C. Deployment of Artificial Intelligence for Radiographic Diagnosis of Covid‐19 Pneumonia in the Emergency Department. J Am Coll Emerg Physicians Open 2020 Dec;1(6):1459–1464. doi: 10.1002/emp2.12297

4. Cha E, Elguindi S, Onochie I, Gorovets D, Deasy JO, Zelefsky M, Gillespie EF. Clinical Implementation of Deep Learning Contour Autosegmentation for Prostate Radiotherapy. Radiother Oncol 2021;159:1–7. doi: 10.1016/j.radonc.2021.02.040

5. Cheikh AB, Gorincour G, Nivet H, May J, Seux M, Calame P, Thomson V, Delabrousse E, Crombé A. How Artificial Intelligence Improves Radiological Interpretation in Suspected Pulmonary Embolism. Eur Radiol 2022 Mar 22;32(9):5831–5842. doi: 10.1007/s00330-022-08645-2

6. Chen W, Wu J, Wei R, Wu S, Xia C, Wang D, Liu D, Zheng L, Zou T, Li R, Qi X, Zhang X. Improving the Diagnosis of Acute Ischemic Stroke on Non-Contrast Ct Using Deep Learning: A Multicenter Study. Insights Imaging 2022 Dec;13(1):184. doi: 10.1186/s13244-022-01331-3

7. Conant EF, Toledano AY, Periaswamy S, Fotin SV, Go J, Boatsman JE, Hoffmeister JW. Improving Accuracy and Efficiency with Concurrent Use of Artificial Intelligence for Digital Breast Tomosynthesis. Radiol Artif Intell 2019 Jul;1(4):e180096. doi: 10.1148/ryai.2019180096

8. Davis MA, Rao B, Cedeno PA, Saha A, Zohrabian VM. Machine Learning and Improved Quality Metrics in Acute Intracranial Hemorrhage by Noncontrast Computed Tomography. Curr Probl Diagn Radiol 2022 Jul;51(4):556–561. doi: 10.1067/j.cpradiol.2020.10.007

9. Diao K, Chen Y, Liu Y, Chen B, Li W, Zhang L, YL Q, Zhang T, Zhang Y, Wu M, Li K, Song B. Diagnostic Study on Clinical Feasibility of an AI-Based Diagnostic System as a Second Reader on Mobile CT Images: A Preliminary Result. Ann Transl Med 2022 Jun;10(12):668. doi: 10.21037/atm-22-2157

10. Duron L, Ducarouge A, Gillibert A, Lainé J, Allouche C, Cherel N, Zhang Z, Nitche N, Lacave E, Pourchot A, Felter A, Lassalle L, Regnard N-E, Feydy A. Assessment of an AI Aid in Detection of Adult Appendicular Skeletal Fractures by Emergency Physicians and Radiologists: A Multicenter Cross-sectional Diagnostic Study. Radiology 2021 Jul;300(1):120–129. doi: 10.1148/radiol.2021203886

11. Elijovich L, Dornbos III D, Nickele C, Alexandrov A, Inoa-Acosta V, Arthur AS, Hoit D. Automated Emergent Large Vessel Occlusion Detection by Artificial Intelligence Improves Stroke Workflow in a Hub and Spoke Stroke System of Care. J NeuroInterventional Surg 2022 Jul;14(7):704–708. doi: 10.1136/neurintsurg-2021-017714

12. Ginat D. Implementation of Machine Learning Software on the Radiology Worklist Decreases Scan View Delay for the Detection of Intracranial Hemorrhage on CT. Brain Sci D. Ginat, Department of Radiology, University of Chicago, Chicago, IL 60615, United States. E-mail: dginat@radiology.bsd.uchicago.edu Switzerland: MDPI; 2021;11(7):832. doi: 10.3390/brainsci11070832

13. Hassan A, Ringheanu V, Tekle W. The Implementation of Artificial Intelligence Significantly Reduces Door-in-Door-Out Times in a Primary Care Center Prior to Transfer. Interv Neuroradiol J Peritherapeutic Neuroradiol Surg Proced Relat Neurosci 2022 Aug;15910199221122848. doi: 10.1177/15910199221122848

14. Jones CM, Danaher L, Milne MR, Tang C, Seah J, Oakden-Rayner L, Johnson A, Buchlak QD, Esmaili N. Assessment of the Effect of a Comprehensive Chest Radiograph Deep Learning Model on Radiologist Reports and Patient Outcomes: A Real-World Observational Study. BMJ Open 2021 Dec;11(12):e052902. doi: 10.1136/bmjopen-2021-052902

15. Ladabaum U, Shepard J, Weng Y, Desai M, Singer SJ, Mannalithara A. Computer-Aided Detection of Polyps Does Not Improve Colonoscopist Performance in a Pragmatic Implementation Trial. Gastroenterology 2023 Mar;164(3). doi: 10.1053/j.gastro.2022.12.004

16. Levy I, Bruckmayer L, Klang E, Ben-Horin S, Kopylov U. Artificial Intelligence-Aided Colonoscopy Does Not Increase Adenoma Detection Rate in Routine Clinical Practice. Am J Gastroenterol 2022 Nov;117(11):1871–1873. doi: 10.14309/ajg.0000000000001970

17. Marwaha A, Chitayat D, Meyn M, Mendoza-Londono R, Chad L. The Point-of-Care Use of a Facial Phenotyping Tool in the Genetics Clinic: Enhancing Diagnosis and Education with Machine Learning. Am J Med Genet A 2021 Apr;185(4):1151–1158. doi: 10.1002/ajmg.a.62092

18. Mueller FC, Raaschou H, Akhtar N, Brejnebol M, Collatz L, Andersen MB. Impact of Concurrent Use of Artificial Intelligence Tools on Radiologists Reading Time: A Prospective Feasibility Study. Acad Radiol 2022;29(7):1085–1090. doi: 10.1016/j.acra.2021.10.008

19. Nehme F, Coronel E, Barringer D, Romero L, Shafi M, Ross W, Ge P. Performance and Attitudes Toward Real-time Computer-aided Polyp Detection during Colonoscopy in a Large Tertiary Referral Center in the United States. Gastrointest Endosc 2023 Feb; doi: 10.1016/j.gie.2023.02.016

20. Oppenheimer J, Lüken S, Hamm B, Niehues S. A Prospective Approach to Integration of AI Fracture Detection Software in Radiographs into Clinical Workflow. Life Basel Switz 2023 Jan;13(1). doi: 10.3390/life13010223

21. Pierce J, Rosipko B, Youngblood L, Gilkeson R, Gupta A, Bittencourt L. Seamless Integration of Artificial Intelligence Into the Clinical Environment: Our Experience With a Novel Pneumothorax Detection Artificial Intelligence Algorithm. J Am Coll Radiol JACR 2021 Nov;18(11):1497–1505.

22. Potretzke T, Korfiatis P, Blezek D, Edwards M, Klug J, Cook C, Gregory A, Harris P, Chebib F, Hogan M, Torres V, Bolan C, Sandrasegaran K, Kawashima A, Collins J, Takahashi N, Hartman R, Williamson E, King B, Callstrom M, Erickson B, Kline T. Clinical Implementation of an Artificial Intelligence Algorithm for Magnetic Resonance-Derived Measurement of Total Kidney Volume. Mayo Clin Proc 2023 May;98(5):689–700. doi: 10.1016/j.mayocp.2022.12.019

23. Quan SY, Wei MT, Lee J, Mohi-Ud-Din R, Mostaghim R, Sachdev R, Siegel D, Friedlander Y, Friedland S. Clinical Evaluation of a Real-Time Artificial Intelligence-Based Polyp Detection System: A US Multi-Center Pilot Study. Sci Rep 2022 Apr 21;12(1):6598. doi: 10.1038/s41598-022-10597-y

24. Raya-Povedano JL, Romero-Martín S, Elías-Cabot E, Gubern-Mérida A, Rodríguez-Ruiz A, Álvarez-Benito M. AI-based Strategies to Reduce Workload in Breast Cancer Screening with Mammography and Tomosynthesis: A Retrospective Evaluation. Radiology 2021 Jul;300(1):57–65. doi: 10.1148/radiol.2021203555

25. Ruamviboonsuk P, Tiwari R, Sayres R, Nganthavee V, Hemarat K, Kongprayoon A, Raman R, Levinstein B, Liu Y, Schaekermann M, Lee R, Virmani S, Widner K, Chambers J, Hersch F, Peng L, Webster DR. Real-Time Diabetic Retinopathy Screening by Deep Learning in a Multisite National Screening Programme: A Prospective Interventional Cohort Study. Lancet Digit Health 2022 Apr;4(4). doi: 10.1016/S2589-7500(22)00017-6

26. Sandbank J, Bataillon G, Nudelman A, Krasnitsky I, Mikulinsky R, Bien L, Thibault L, Albrecht Shach A, Sebag G, Clark D, Laifenfeld D, Schnitt S, Linhart C, Vecsler M, Vincent-Salomon A. Validation and Real-World Clinical Application of an Artificial Intelligence Algorithm for Breast Cancer Detection in Biopsies. Npj Breast Cancer 2022 Dec;8(1):129. doi: 10.1038/s41523-022-00496-w

27. Schmuelling L, Franzeck FC, Nickel CH, Mansella G, Bingisser R, Schmidt N, Stieltjes B, Bremerich J, Sauter AW, Weikert T, Sommer G. Deep Learning-Based Automated Detection of Pulmonary Embolism on CT Pulmonary Angiograms: No Significant Effects on Report Communication Times and Patient Turnaround in the Emergency Department Nine Months After Technical Implementation. Eur J Radiol 2021 Aug;141:109816. doi: 10.1016/j.ejrad.2021.109816

28. Seyam M, Weikert T, Sauter A, Brehm A, Psychogios M-N, Blackham KA. Utilization of Artificial Intelligence-based Intracranial Hemorrhage Detection on Emergent Noncontrast CT Images in Clinical Workflow. Radiol Artif Intell 2022;4(2):e210168.

29. Tchou PM, Haygood TM, Atkinson EN, Stephens TW, Davis PL, Arribas EM, Geiser WR, Whitman GJ. Interpretation Time of Computer-aided Detection at Screening Mammography. Radiology 2010 Oct;257(1):40–46. doi: 10.1148/radiol.10092170

30. Tricarico D, Calandri M, Barba M, Piatti C, Geninatti C, Basile D, Gatti M, Melis M, Veltri A. Convolutional Neural Network-Based Automatic Analysis of Chest Radiographs for the Detection of COVID-19 Pneumonia: A Prioritizing Tool in the Emergency Department, Phase I Study and Preliminary “Real Life” Results. Diagnostics 2022;12(3):570. doi: https://dx.doi.org/10.3390/diagnostics12030570 PT - Article

31. Vassallo L, Traverso A, Agnello M, Bracco C, Campanella D, Chiara G, Fantacci ME, Lopez Torres E, Manca A, Saletta M, Giannini V, Mazzetti S, Stasi M, Cerello P, Regge D. A Cloud-Based Computer-Aided Detection System Improves Identification of Lung Nodules on Computed Tomography Scans of Patients with Extra-Thoracic Malignancies. Eur Radiol 2019 Jan;29(1):144–152. doi: 10.1007/s00330-018-5528-6

32. Wang P, Berzin TM, Glissen Brown JR, Bharadwaj S, Becq A, Xiao X, Liu P, Li L, Song Y, Zhang D, Li Y, Xu G, Tu M, Liu X. Real-Time Automatic Detection System Increases Colonoscopic Polyp and Adenoma Detection Rates: A Prospective Randomised Controlled Study. Gut 2019 Oct;68(10):1813–1819. doi: 10.1136/gutjnl-2018-317500

33. Wang M, Xia C, Huang L, Xu S, Qin C, Liu J, Cao Y, Yu P, Zhu T, Zhu H, Wu C, Zhang R, Chen X, Wang J, Du G, Zhang C, Wang S, Chen K, Liu Z, Xia L, Wang W. Deep Learning-Based Triage and Analysis of Lesion Burden for Covid-19: A Retrospective Study with External Validation. Lancet Digit Health 2020 Oct;2(10):e506–e515. doi: 10.1016/S2589-7500(20)30199-0

34. Wittenberg R, Berger FH, Peters JF, Weber M, van Hoorn F, Beenen LFM, van Doorn MMAC, van Schuppen J, Zijlstra IjAJ, Prokop M, Schaefer-Prokop CM. Acute Pulmonary Embolism: Effect of a Computer-assisted Detection Prototype on Diagnosis—An Observer Study. Radiology 2012 Jan;262(1):305–313. doi: 10.1148/radiol.11110372

35. Wong J, Huang V, Wells D, Giambattista J, Giambattista J, Kolbeck C, Otto K, Saibishkumar EP, Alexander A. Implementation of Deep Learning-Based Auto-Segmentation for Radiotherapy Planning Structures: A Workflow Study at Two Cancer Centers. Radiat Oncol 2021 Dec;16(1):101. doi: 10.1186/s13014-021-01831-4

36. Wong K, Homer S, Wei S, Yaghmai N, Estrada Paz O, Young T, Buhr R, Barjaktarevic I, Shrestha L, Daly M, Goldin J, Enzmann D, Brown M. Integration and Evaluation of Chest X-Ray Artificial Intelligence in Clinical Practice. J Med Imaging 2023 Sep;10(5):051805. doi: 10.1117/1.JMI.10.5.051805

37. Yang Y, Pan J, Yuan M, Lai K, Xie H, Ma L, Xu S, Deng R, Zhao M, Luo Y, Lin X. Performance of the AIDRScreening System in Detecting Diabetic Retinopathy in the Fundus Photographs of Chinese Patients: A Prospective, Multicenter, Clinical Study. Ann Transl Med 2022 Oct;10(20):1088. doi: 10.21037/atm-22-350

38. Zia A, Fletcher C, Bigwood S, Ratnakanthan P, Seah J, Lee R, Kavnoudias H, Law M. Retrospective Analysis and Prospective Validation of an Ai-Based Software for Intracranial Haemorrhage Detection at a High-Volume Trauma Centre. Sci Rep 2022 Nov;12(1):19885. doi: 10.1038/s41598-022-24504-y
