# Supplementary material for: Facilitators and Barriers to Implementing AI in Routine Medical Imaging: Systematic Review and Qualitative Analysis
Source: J Med Internet Res. 2025 Jul 21;27:e63649. doi: 10.2196/63649 (PMC12322614; doi:10.2196/63649)
Supplement: Multimedia Appendix 9 [file jmir_v27i1e63649_app9.docx]

### **Multimedia Appendix 9. ChatGPT transcript.**

**You:**

Can you mark in this text were the tense varies? All 38 included studies examined various effects stemming from the implementation of AI tools in medical imaging tasks. 31 studies (82.6%) reported efficiency outcomes with 22 studies showing enhanced efficiency, while 2 studies reported negative effects, and 7 studies indicated no changes in efficiency. Five studies assessed the impact of AI on workload or required work steps, with four reporting reductions and one indicating an increase. 16 of the included studies (42.1%) reported on the performance of AI solutions, in terms of changes in detection rates, need for human oversight, or quality of the AI-based results. 13 studies (34.2%) also discussed outcomes for patients such as enhanced safety or quality control due to AI, a reduced time to diagnosis or treatment, prolonged stay in the ED, and increased detection rates possibly leading to additional unnecessary treatments or increased workload [66]. Full details on reported study outcomes can be found in Supplementary Material 5. 11 studies (29.0%) used some form of survey or questionnaire to gather clinicians’ opinions and experiences. Most commonly they used clinicians’ self-reports on AIs’ impact on the diagnosis and on efficiency, followed by their attitude towards AI, their satisfaction or usefulness as well as the usability of the AI tool. Notably only the study of Jones et al. used an established tool, i.e., the Systems Usability Scale [49]. Full details can be found Supplementary Material 6.

**ChatGPT:**

Sure, here are the instances where the tense varies in the text:

1. "All 38 included studies examined various effects stemming from the implementation of AI tools in medical imaging tasks." - Past tense ("examined").
2. "31 studies (82.6%) reported efficiency outcomes with 22 studies showing enhanced efficiency, while 2 studies reported negative effects, and 7 studies indicated no changes in efficiency." - Past tense ("reported").
3. "Five studies assessed the impact of AI on workload or required work steps, with four reporting reductions and one indicating an increase." - Past tense ("assessed", "reported", "indicating").
4. "16 of the included studies (42.1%) reported on the performance of AI solutions, in terms of changes in detection rates, need for human oversight, or quality of the AI-based results." - Past tense ("reported").
5. "13 studies (34.2%) also discussed outcomes for patients such as enhanced safety or quality control due to AI, a reduced time to diagnosis or treatment, prolonged stay in the ED, and increased detection rates possibly leading to additional unnecessary treatments or increased workload [66]." - Past tense ("discussed").
6. "11 studies (29.0%) used some form of survey or questionnaire to gather clinicians’ opinions and experiences." - Past tense ("used").
7. "Most commonly they used clinicians’ self-reports on AIs’ impact on the diagnosis and on efficiency, followed by their attitude towards AI, their satisfaction or usefulness as well as the usability of the AI tool." - Past tense ("used").
8. "Notably only the study of Jones et al. used an established tool, i.e., the Systems Usability Scale [49]." - Past tense ("used").

**You:**

Can you make this more reader friendly and concise? Overall, we saw that the importance of different dimensions of facilitators and barriers varied between the different level of implementation, but there was always a variety of dimension involved, highlighting that strong interrelations between various factors coming together when implementing AI into healthcare.

**ChatGPT:**

Certainly! Here's a more reader-friendly and concise version:

"In our analysis, we found that the significance of various facilitators and barriers differed across different stages of implementation. However, there was consistently a diverse range of factors at play, emphasizing the complex interplay of various elements when integrating AI into healthcare."

**You:**

Can you make this more concise? Our analysis revealed more facilitators than barriers reported in the studies, which we clustered in dimensions with different themes due to the same topic being mentioned positively or negatively, like AI being easy or not easy to use. The Evaluation of AI Use dimension, particularly the Usefulness theme, was most frequently mentioned, reflecting the studies’ focus on AI evaluation in clinical practice. This also highlights the importance of AIs’ perceived usefulness to the users evaluation and adoption when implementing it into the workflow [28]. Themes like Explainability of AI or Stakeholder Involvement were mostly facilitators, with only a few studies identifying them as a barrier. Medicolegal Concerns was exclusively mentioned as a barrier, highlighting healthcare providers' uncertainty about AI use. This matches current efforts of regulatory bodies like the European Commission and FDA which are increasingly addressing this issue [73].

**ChatGPT:**

Our analysis found more facilitators than barriers, which we grouped into thematic dimensions. The Evaluation of AI Use dimension, particularly the Usefulness theme, was most commonly cited, underscoring its importance in clinical practice. This emphasizes the significance of perceived usefulness in the evaluation and adoption of AI in workflows. Themes like Explainability of AI or Stakeholder Involvement were predominantly facilitators, with few studies noting them as barriers. Medicolegal Concerns emerged solely as a barrier, reflecting healthcare providers' uncertainties, aligning with ongoing regulatory efforts by bodies like the European Commission and FDA.

**You:**

Can you make this less technical and make clear that there is then the challenge to tackle when implementng ai, which means we compiled a list of factors which are very relevant to AI implementation? Our analysis found more facilitators than barriers, which we grouped into dimensions. Within the dimensions we clustered recurring themes, which were depending on the study mentioned as a facilitator or barrier due to the same theme being mentioned positively or negatively, like AI being easy or not easy to use

**ChatGPT:**

Our examination uncovered more things helping than hindering AI implementation. We organized these into categories, where recurring themes emerged. These themes were seen differently in studies — sometimes as a help, sometimes as a hurdle. For instance, ease of AI use could be seen positively or negatively depending on the study.

**You:**

Can you make this better understandable and more cocise? Our analysis uncovered that extracted themes were seen differently in studies — sometimes as a facilitator, sometimes as a barrier. For instance, ease of AI use could be seen positively or negatively depending on the study. The description of the theme either as a help or hurdle was mostly depending on the presence vs. absence or the fulfilment vs. non-fulfilment of that theme in the study, for example AI use being distraction vs non-distracting. Therefore our study results in a compiled list of factors which are necessary to consider during AI implementation.

**ChatGPT:**

Our analysis revealed that themes extracted from the studies were perceived differently — sometimes as helpful, other times as obstacles. For instance, ease of AI use could be viewed positively or negatively depending on the study. Whether a theme was seen as beneficial or challenging often depended on whether it was present or absent, or fulfilled or unfulfilled in the study, such as AI use being either distracting or non-distracting. Consequently, our study provides a compiled list of factors essential for consideration during AI implementation.

**You:**

give me synonyms for rare

**ChatGPT:**

Sure, here are some synonyms for "rare":

1. Uncommon
2. Infrequent
3. Scarce
4. Unusual
5. Sparse
6. Limited
7. Scant
8. Occasional
9. Seldom
10. Few and far between

**You:**

Can you make this more concise and better readable?

The focus of this network aligns with the theoretical background, the SEIPS model, which places people at the center and emphasizes the human-centered design [16]. This also matches the focus of these studies which initially implement AI to evaluate and test how the AI solution fits within their work system and which measures need to be taken to optimize the fit. In the field of AI adoption there is a growing awareness for human-centered design and the importance of stakeholder involvement in AI implementation in healthcare, which is supported by our results

**ChatGPT:**

This network's focus is in line with the SEIPS model, which prioritizes people and human-centered design [16]. This resonates with studies that use AI to assess its integration into work systems and determine necessary optimizations. There's a rising acknowledgment in AI adoption of the significance of human-centered design and stakeholder engagement in healthcare, supported by our findings.

**You:**

Can you make this better readable?

This systematic review synthesizes reported facilitators and barriers to AI adoption in medical imaging, revealing the importance of AI’s usefulness and its fit into the workflow. Most studies did not directly report facilitators and barriers which highlights the need for comprehensive reporting of these factors to facilitate learning across studies. Our findings show a strong focus of the included on technological features emphasizing our call for a holistic and human-centric research to AI implementation impediment for AI to optimally support healthcare.

**ChatGPT:**

This systematic review examines facilitators and barriers to AI adoption in medical imaging, emphasizing the significance of AI's utility and its integration into workflows. Many studies didn't directly address these factors, underscoring the necessity for comprehensive reporting to foster knowledge sharing. Our findings reveal a predominant focus on technological aspects, underscoring the need for holistic, human-centric research to fully leverage AI's potential in healthcare.

**You:**

Can you make this more concise and better readable?

The use of artificial intelligence (AI)-based solutions in healthcare is advancing rapidly, especially in medical imaging, due to its potential for enhancing efficiency and reducing workload. When being implemented into clinical workflows, numerous facilitators or barriers can influence AI adoption. We aimed to assess these facilitators and barriers to AI implementation that were reported in studies who evaluated AI solutions in medical imaging clinical practice.

**ChatGPT:**

Sure, here's a more concise and clearer version:

"AI is rapidly advancing in healthcare, particularly in medical imaging, offering improved efficiency and reduced workload. We examined factors influencing AI adoption in clinical workflows through studies evaluating AI solutions in medical imaging."

**You:**

Can you make this better readable?

We conducted a systematic literature review, searching six medical databases. We extracted key characteristics, study outcomes and as well as moderators influencing the use of AI. Two reviewers performed a qualitative content analysis to extract and categorize reported facilitators and barriers to AI implementation. An Epistemic Network Analysis was applied to examine the interrelationships between facilitators and barriers.

**ChatGPT:**

Certainly, here's a more readable version:

"We conducted a systematic literature review across six medical databases. We extracted key characteristics, study outcomes, and factors influencing AI use. Two reviewers conducted qualitative content analysis to categorize reported facilitators and barriers to AI implementation. We then applied Epistemic Network Analysis to examine the interrelationships between these factors."

**You:**

can you make it a bit more concise?

**ChatGPT:**

Of course:

"We conducted a systematic review across six medical databases, extracting key characteristics, outcomes, and factors influencing AI use. Two reviewers analyzed and categorized facilitators and barriers to AI implementation. Epistemic Network Analysis was then applied to explore their interrelationships."

**You:**

Can you make this better readable and more concise?

We screened 13,760 records and included 38 articles in the final review. We identified a total of 12 overarching dimensions with 37 subthemes of factors influencing the implementation of AI in healthcare workflows. Most of these themes were categorized both as facilitators and barriers, depending on the study context. Dimensions named most frequently were the Evaluation of AI Use and the Fit into the Workflow. Importance and connections between different factors varied depending on how integrated the AI tool was into the routine workflow. Notably, many studies focused on performance metrics and the evaluation of objective measures, rather than clinicians’ experiences or outcomes.

**ChatGPT:**

We reviewed 13,760 records, ultimately including 38 articles. We identified 12 dimensions and 37 subthemes influencing AI implementation in healthcare workflows. Many themes served as both facilitators and barriers, depending on context. Key dimensions included Evaluation of AI Use and Fit into Workflow. Importance and connections varied based on AI integration. Studies often prioritized performance metrics over clinician experiences or outcomes.

**You:**

Can you make this better understandable?

Regarding facilitators and barriers of AI implementation we faced a broad variety with the same topic being reported as having a positive or negative effect depending on the study. Therefore we coded dimensions within the documents and later clustered the single statements into themes named within the different dimensions, while noting separately whether the statement described a facilitator and barrier.

**ChatGPT:**

In exploring the facilitators and barriers of AI implementation, we encountered a wide range of factors. Interestingly, the same topic could be reported as both positive and negative depending on the study context. To manage this complexity, we coded dimensions within the documents and then grouped individual statements into themes based on these dimensions. Importantly, we distinguished whether each statement described a facilitator or a barrier.

**You:**

Can you put this in english title-case?

Fit into the workflow

**ChatGPT:**

"Fit Into the Workflow"

**You:**

Can you correct this?

AI Impact on Clinicians Role

**ChatGPT:**

"AI Impact on Clinicians' Role"
